# A Molecular Probe for the Detection of Polar Lipids in Live Cells

**DOI:** 10.1371/journal.pone.0161557

**Published:** 2016-08-23

**Authors:** Christie A. Bader, Tetyana Shandala, Elizabeth A. Carter, Angela Ivask, Taryn Guinan, Shane M. Hickey, Melissa V. Werrett, Phillip J. Wright, Peter V. Simpson, Stefano Stagni, Nicolas H. Voelcker, Peter A. Lay, Massimiliano Massi, Sally E. Plush, Douglas A. Brooks

**Affiliations:** 1 School of Pharmacy and Medical Science, University of South Australia, Adelaide, South Australia, Australia; 2 Vibrational Spectroscopy Core Facility, The University of Sydney, Sydney, New South Wales, Australia; 3 Future Industries Institute, University of South Australia, Mawson Lakes, South Australia, Australia; 4 Department of Chemistry and Nanochemistry Research Institute, Curtin University, Bentley, Western Australia, Australia; 5 Department of Industrial Chemistry “Toso Montanari”, University of Bologna, Bologna, Italy; Louisiana State University Health Sciences Center, UNITED STATES

## Abstract

Lipids have an important role in many aspects of cell biology, including membrane architecture/compartment formation, intracellular traffic, signalling, hormone regulation, inflammation, energy storage and metabolism. Lipid biology is therefore integrally involved in major human diseases, including metabolic disorders, neurodegenerative diseases, obesity, heart disease, immune disorders and cancers, which commonly display altered lipid transport and metabolism. However, the investigation of these important cellular processes has been limited by the availability of specific tools to visualise lipids in live cells. Here we describe the potential for ReZolve-L1^™^ to localise to intracellular compartments containing polar lipids, such as for example sphingomyelin and phosphatidylethanolamine. In live *Drosophila* fat body tissue from third instar larvae, ReZolve-L1^™^ interacted mainly with lipid droplets, including the core region of these organelles. The presence of polar lipids in the core of these lipid droplets was confirmed by Raman mapping and while this was consistent with the distribution of ReZolve-L1^™^ it did not exclude that the molecular probe might be detecting other lipid species. In response to complete starvation conditions, ReZolve-L1^™^ was detected mainly in Atg8-GFP autophagic compartments, and showed reduced staining in the lipid droplets of fat body cells. The induction of autophagy by Tor inhibition also increased ReZolve-L1^™^ detection in autophagic compartments, whereas Atg9 knock down impaired autophagosome formation and altered the distribution of ReZolve-L1^™^. Finally, during *Drosophila* metamorphosis fat body tissues showed increased ReZolve-L1^™^ staining in autophagic compartments at two hours post puparium formation, when compared to earlier developmental time points. We concluded that ReZolve-L1^™^ is a new live cell imaging tool, which can be used as an imaging reagent for the detection of polar lipids in different intracellular compartments.

## Introduction

Lipids are essential organic components of cells that can be used in membrane formation, intracellular signalling and to provide energy for cellular function [1]. Structurally, glycerolipids/phospholipids are the primary components of biological membranes [2], and sphingolipids and sterols can form micro-domains within these cellular membranes to facilitate protein interactions [3–7] and intracellular signalling [8]. The *de novo* synthesis of lipids begins with the production of fatty acids from acetyl-CoA *via* a series of enzymatic steps [2]. Fatty acids can then be converted into neutral lipids, which can be stored for later use in energy production, or synthesised into structural or signalling lipids, such as phospholipids [2]. Lipid droplets are the main site of lipid storage in cells and are reported to contain a core of neutral lipids comprised mainly of triacylglycerol and esterified sterols that are surrounded by an outer phospholipid monolayer [9, 10]. This limiting membrane of the lipid droplet is thought to be enriched with polar lipids, such as phospholipids, cholesterol and fatty acids, which can form functional micro-domains [11, 12]. While this clear demarcation of lipid families in lipid droplets is commonly presented in the literature, it can be expected that in reality the lipid droplet is far more heterogenous and complex in its biology.

Lipids that are stored within the core of lipid droplets can be mobilised in response to cellular demand and this often involves enzymatic modification by lipases. In lipid droplets, for example, adipose triglyceride lipase, hormone-sensitive lipase and monoacylglycerol lipase function sequentially to hydrolyse triacylglycerol to fatty acids and glycerol, while sterol esters can also be hydrolysed by hormone-sensitive lipases [13]. Sterol esters and triacylglycerol can also be hydrolysed by lysosomal degradation. This can involve a process called macroautophagy (herein referred to as autophagy), where the lipids are sequestered from lipid droplets to autophagosomes and then interact with acid lipase in endosomes and lysosomes as the autophagosomes undergo maturation to form autolysosomes [14]. Autophagy has been strongly implicated in the maintenance of cellular lipid homeostasis, trafficking, efflux and metabolism [15–18]. Autophagy also has an important role in controlling the amount of sphingolipids, gangliosides, sterols and phospholipids in cells and can modulate the trafficking, degradation and membrane composition of these lipids [14, 19–21]. In turn, lipids can regulate the induction of autophagy via interactions with the autophagic regulator, target of rapamycin (TOR) [22–24]; placing autophagy as a key regulator of lipid homeostasis [25–27]. However, a major limitation in the fields of lipid biology and autophagy has been the ability to visualise these important cellular processes in live cells.

Visualisation of lipids is difficult in live cells [28], as many of the commonly used stains require sample fixation, such as Filipin III, Oil Red O and Nile Red [29–33]. Cell fixation can compromise cellular ultrastructure [28, 34] and is particularly problematic for lipids, especially when using alcohol-based solvents or fixatives, as they can cause the fusion of lipid droplets and/or extraction of lipids from cell and tissue samples [9, 35–37]. Neutral lipid dyes such as BODIPY^®^(493/503), LipidTOX and LD540 can be used for lipid imaging in live or fixed samples and have overcome some of the solubility issues encountered with lipid dyes [32, 38, 39]; however, fluorescent probes for polar lipids are still somewhat limited. Filipin III, which is widely used for the detection of cholesterol, is one of the few stains available for the detection of polar lipids. The use of Filipin III, however, requires cell fixation, additionally its mode of interaction is not well defined and it has recently been shown to have high affinity for GM1 and GM2 gangliosides, as well as cholesterol [8, 39, 40]. A wide range of fluorescently labelled lipid analogues are available for live cell imaging of lipid trafficking events, but their behaviour does not always match that of the endogenous lipids. Thus, there is an unmet need for new lipophilic dyes that can be used in live cell imaging.

ReZolve-L1^™^, a lipophilic, tricarbonyl rhenium(I) diimine luminescent molecular probe, has recently become commercially available (Rezolve Scientific, Adelaide, Australia) and has the capacity to image lipid droplets in live cells ([41] reported as complex 1). ReZolve-L1^™^ is a phosphorescent transition metal complex that exhibits a long excitation half-life, in the order of hundreds of nanoseconds, long photostability, and has a large Stokes shift of *ca* 200 nm, which are ideal features for cellular imaging [42]. ReZolve-L1^™^ can be used with two photon microscopy allowing flexibility of sample preparation as well as less cell damage and greater sample penetration, due to the use of lower energy excitation (NB. ReZolve-L1^™^ can also be excited using standard methods of fluorescence microscopy, with for example a UV light source or a 405nm laser). Here we show that ReZolve-L1^™^ has an affinity for polar lipids in two pseudo cellular models. Using Raman spectroscopic mapping [43], we show that localisation can be more closely correlated with polar lipids and not signals from esterified lipids. Interestingly, our data also supports the premise that lipid droplets are of a more heterogeneous nature than is classically accepted. Using *Drosophila* as a model system [44–50], we show that ReZolve-L1^™^ is detected in lipid droplets, but under complete starvation conditions and during larval to adult metamorphosis, is mainly detected in autophagic compartments. Furthermore, ReZolve-L1^™^ was found to be compatible with different fluorescent reporters for multi-colour live cell imaging, giving a broad application base in cell biology.

## Results

### ReZolve-L1^™^ interacts with polar lipids.

The ‘specificity’ of dyes that are employed to label lipid droplets (e.g. Nile Red or BODIPY 493/503) is commonly related to high dye lipophilicity and a preference of the dye for non-polar lipids such as cholesterol esters (CE) and triacylglcerides (TAG) [51, 52]. The staining of these dyes is predominantly limited to the lipid droplets. While ReZolve-L1^™^ has been shown to predominantly label lipid droplets, detection of the dye was not limited to lipid droplets. This suggests that ReZolve-L1^™^ may show a different lipid affinity in comparison to other lipid ‘specific’ probes. Interestingly, while ReZolve-L1^™^ is reasonably lipophilic (log *P* value = 2.53 ± 0.08), both BODIPY 493/503 (log *P* = 3.5 ± 0.04 [38]) and Nile Red (log *P* = 5.0 [53]) are significantly more lipophilic. Therefore, the rationale for accumulation of ReZolve-L1^™^ in lipid droplets is not one purely of lipophilicity; and accumulation may be attributed to an affinity for certain lipid species.

**Fig 1:**
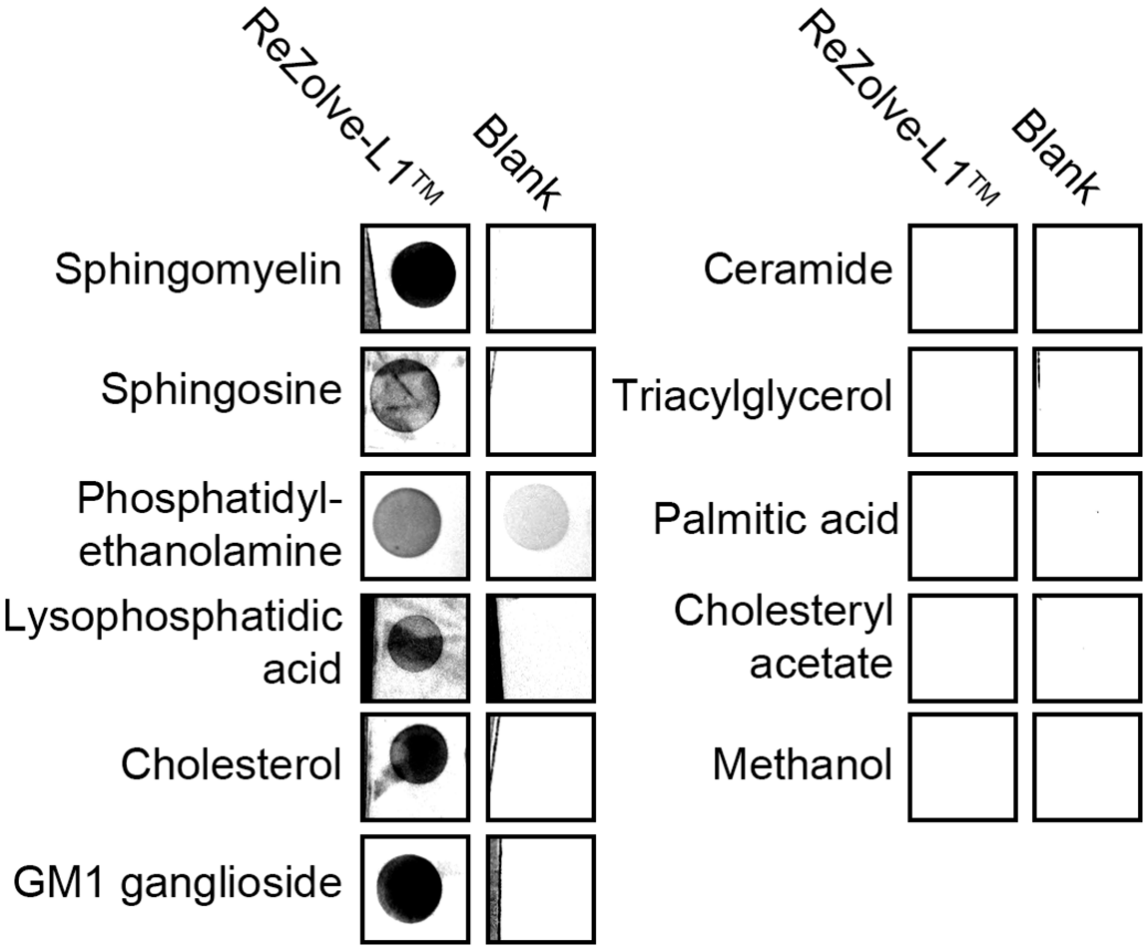
ReZolve-L1^™^ interacts with polar lipids. A ReZolve-L1^™^ lipid overlay demonstrates an interaction between the molecular probe and six polar lipid species (left hand side of the Fig) and no detectable interaction with either four neutral lipid species, fatty acid species or methanol controls (right hand side of the Fig). Blanks show the detection of the lipid background, without incubation with ReZolve-L1^™^.

To determine if the accumulation of ReZolve-L1^™^ in lipid droplets is related to an affinity for different lipid species, lipid overlay experiments were performed using ten lipids, representing lipid groups commonly found in cells. In these lipid overlay experiments ReZolve-L1^™^ interacted with sphingomyelin, sphingosine, phosphatidylethanolamine, lysophosphatidic acid, cholesterol and GM1 ganglioside (Fig 1). There was no detectable interaction of ReZolve-L1^™^ with either ceramide, triacylglycerol’s (a mixture containing 5 species was tested), fatty acid species, palmitic acid or cholesteryl acetate (Fig 1). To ensure that the lipids were not removed from the polyvinylidene difluoride (PVDF) membranes during processing, the lipid overlay experiments were carried out in triplicate and repeated using three different washing protocols. These washing protocols involved either cold 10% ethanol, methanol or PBS, in each instance consistent results were obtained, which indicated that lipid removal from the PVDF membranes was not a likely contributing factor for the observed interactions. These lipid overlay experiments combined with the measured log *P* value suggest that the accumulation of ReZolve-L1^™^ in lipid droplets cannot primarily be attributed to an affinity for CE and TAG’s [52].

Lipid overlay experiments only provide a qualitative indication that ReZolve-L1 has an affinity for lipid species, but this was not representative of the fluid nature in which lipids exist within cells. Liposomes formed from polar lipids offer the opportunity to study lipids in a more fluid-like environment; note non-polar lipids such as TAGs do not form liposomes so were not included in this line of enquiry. Therefore, in an effort to confirm that the trend observed in the lipid overlay experiments is representative of ReZolve-L1^™^’s affinity for polar lipids, liposomes were prepared, incubated with ReZolve-L1^™^ and analysed for fluorescence emission. The properties of liposomes can be controlled to a large degree by lipid composition [54], and therefore two preparations were assessed. 1,2-Dimyristoyl-sn-glycero-3-phosphocholine (DMPC) was chosen as it is used extensively as a model liposome system. DMPC and cholesterol (DMPC:cholesterol (8:1 molar ratio)) liposomes were also prepared, as high ratios of cholesterol have been associated with reduced bilayer permeability [55]. Liposomes formed with either DMPC or DMPC:cholesterol were found to be ~100 nm in size as determined by dynamic light scattering (DLS) measurements. The liposomes were then incubated with ReZolve-L1^™^ for a period of 30 minutes, and excess ReZolve-L1^™^ was then removed by a size-exclusion column. Significant fluorescence emission was recorded for both types of liposome preparations, following incubation with ReZolve-L1^™^ and excitation at either 256 nm or 405 nm. Importantly, the emission spectrum recorded from both liposome preparations following incubation with ReZolve-L1^™^ matched that of pure ReZolve-L1^™^ in solution; with no evidence of fluorescence emission observed from control liposomes (see S1 Fig). This confirmed that ReZolve-L1^™^ interacted with polar lipids in a fluid-like environment and that the interaction observed in the lipid overlay experiments was not an artefact of the experimental method.

**Fig 2:**
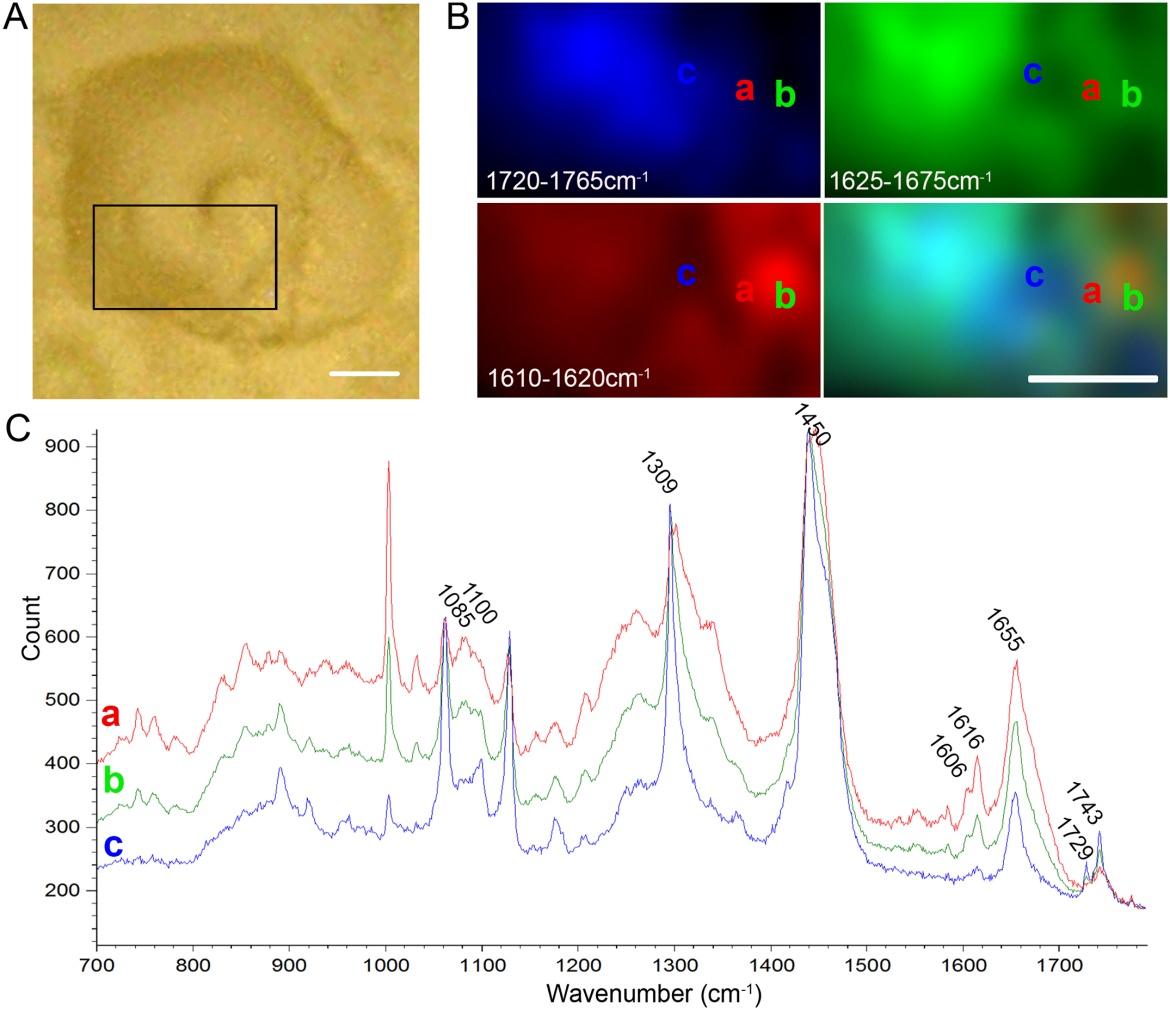
Raman map of ReZolve-L1^™^ in 3T3 adipocytes relative to lipid species. (**A**) Transmitted light image of a 3T3 cell on which Raman mapping was performed. Black box indicates area scanned. (**B**) Raman maps showing the distribution of **ν**(C = O) lipid esters (blue), lipid backbone **ν**(C = C) (green) and ReZolve-L1^™^ (red). Raman maps were generated by calculating the area under the following spectral regions; 1720–1765 cm^-1^, 1625–1675 cm^-1^ and 1610–1620 cm^-1^, respectively. (**C**) Spectra extracted from locations **a, b** and **c** shown in panel (**B**).

The environment of lipids in liposomes is still not particularly reflective of the cellular environment; where lipids are known to be found in close interaction with proteins and a multitude of other lipids (e.g. lipid rafts, caveolin proteins, channel proteins, etc). This type of heterogeneous environment is difficult to represent in a synthetic liposome environment. Therefore, in an effort to understand and investigate what is governing the localisation pattern of ReZolve-L1^™^ in the cell, we chose to use the non-invasive vibrational technique of confocal Raman spectroscopy. Raman spectroscopy can potentially provide information on the localisation of probe accumulation within the lipid droplet in relation to the actual (as opposed to an artificial environment) lipid distribution (e.g. homogeneity vs heterogeneity) in a cellular environment. The mammalian adipocyte 3T3-L1 cell line was chosen, as these cells have large lipid droplets (up to 150 μm) to facilitate the imaging [56]. The 3T3-L1 cells were cultured on calcium fluoride substrate, then rapidly fixed using cold methanol and air dried before being incubated with ReZolve-L1^™^; the use of cold methanol in this manner eliminates biochemical changes [37, 43]. Lipid droplets were then identified by bright field imaging (Fig 2A) and Raman spectra were then collected from the cell centre as determined by the quantity of signal in the z-axis. To illustrate the distribution of lipids within the 3T3-L1 adipocytes the area under the bands at 1655 cm^-1^, **ν**(C = C), and 1743 cm^-1^, **ν**(C = O), were measured for each spectrum collected and plotted versus the spatial co-ordinates from where the data was collected to produce false-colour images (Fig 2). The band at 1655 cm^-1^ is assigned to the **ν**(C = C) with expected contributions from both polar and non-polar lipids and the band at 1743 cm^-1^ is assigned to the **ν**(C = O) which is mainly due to contributions from TAGs and CEs [57]. The same process was employed for a band at 1616 cm^-1^ identified as a key band from the reference spectra of solid ReZolve-L1^™^ (S2A Fig). By overlaying the Raman maps generated from the ReZolve-L1^™^ band (1616 cm^-1^) and the **ν**(C = C) lipid band (1655 cm^-1^), co-localisation was observed between the lipids and ReZolve-L1^™^ (Fig 2B). In contrast, ReZolve-L1^™^ did not completely co-localise with the 1743 cm^-1^ band attributed to neutral lipid species (Fig 2B).

Raman spectra were then extracted from regions within a Raman map of high, **a**, medium, **b**, and low, **c**, intensity of the band at 1616 cm^-1^ (representative spectra can be seen in Fig 2C). The identification of specific molecules from Raman spectra is difficult, however using integrated intensities and the presence of multiple Raman bands associated with key functional groups we can with a degree of confidence ascertain which key bands are associated with ReZolve-L1^™^. For example, while it is known that proteins also appear in this region the lack of the amide II band (1525 cm^-1^) suggests that the band at 1616 cm^-1^ is independent of protein concentration and that ReZolve-L1^™^ is the main contributor to this band. Examination of the transmitted light image (Fig 2A) shows that the regions from which the spectra were extracted are closely associated with the core of the lipid droplet and the limiting membrane. This is further supported by the presence of **ν**(C = O) band (1743cm^-1^) likely to be associated with ester groups.

Close examination of the Raman spectra revealed that an increase in the intensity of the ReZolve-L1^™^ bands (1606 and 1616 cm^-1^) coincided with an increased intensity in the band at 1655 cm^-1^, but were independent of the band 1743cm^-1^, which as stated we have attributed to **ν**(C = O). We have assigned the band at 1655 cm^-1^ to the **ν**(C = C) of lipids and not proteins based on integrated intensities with other known lipid bands present in the spectra and the lack of bands associated with proteins. This further confirmed that the localisation of ReZolve-L1^™^ within or in close approximation to the lipid droplet was not well aligned with the presence of an ester signal (as defined by the **ν**(C = O) band), but was more closely aligned with other lipid species. This lipid preference was further supported by analysis of the second derivative Raman spectrum (S2C Fig). The second derivative of the cell spectra from region **b** (medium ReZolve-L1^™^ content) and **c** (low ReZolve-L1^™^ content) clearly exhibit two **ν**(C = O) bands at 1729 and 1743 cm^-1^, but only the second derivative of the spectrum from **b** also has the two unique ReZolve-L1^™^ bands (S2C Fig). In contrast, spectrum **a** was extracted from a region containing a high ReZolve-L1^™^ content and has no **ν**(C = O) bands detected from the analysis of the second derivative (S2C Fig). This suggested that ReZolve-L1^™^ localisation maybe somewhat distinct from regions with higher ester content, but still aligned with other lipid species.

In spectra that exhibited intense ReZolve-L1^™^ Raman bands (extracted from region **a** and **b**, Fig 2B) a broadening of the band at ~ 1300cm^-1^, attributed to a CH_2_ twisting mode (Fig 2C) together with variation(s) in the spectral region of 1050-1150cm^-1^ was observed. These regions are known to be representative of the presence of polar lipids for example 1050–1150 cm^-1^ is commonly associated with **ν**(C-C) and **ν**(P-O). The presence of these bands combined with the presence of the **ν**(C = O) band in spectra extracted from region **b** suggested that the lipid in lipid droplets may be more heterogeneously distributed than has been commonly described. However, as ReZolve-L1^™^ contributes to signal in these regions it was difficult to assign these peaks to specific biological components with a high level of certainty (Figs 2C and S2C). Taken together these results suggested that while ReZolve-L1^™^ was able to interact with lipids in a cellular environment, the type of lipids involved in this interaction were distinct from esterified forms.

### Polar lipids were detected in lipid droplets of *Drosophila* fat body by ReZolve-L1^™^ and Raman spectroscopy

**Fig 3:**
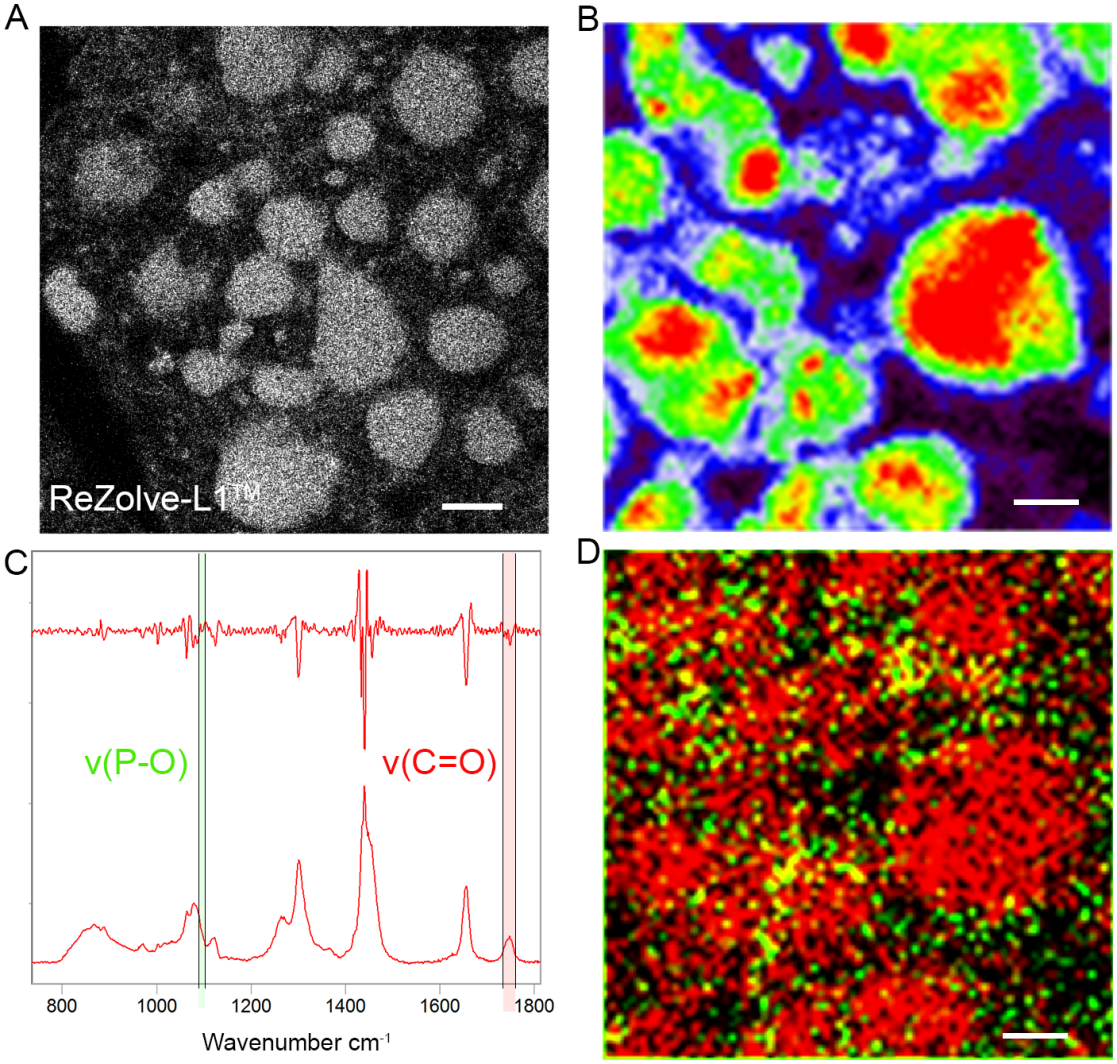
Lipid droplets in *Drosophila* fat body contain polar lipids. (**A**) Confocal micrograph of *Drosophila* fat body tissue from larvae at -4 hours prior to puparium formation (-4 h PF), stained with ReZolve-L1^™^. (**B**) Raman map of lipid droplets of *Drosophila* fat body tissue from larvae at -4 h PF. Map generated based on integration of the **ν**(C = C) region 1625–1675 cm^-1^. (**C**) Representative Raman spectrum form lipid droplet core, with its corresponding second derivative spectrum. Green shading indicates the **ν**(P-O) region, red shading indicates the **ν**(C = O) region. (**D**) Overlaid Raman maps shows the distribution of **ν**(P-O) region (green; 1094–1098 cm^-1^) and the **ν**(C = O) (red;1720–1765 cm^-1^). Scale bar = 10 μm.

To further confirm the link between ReZolve-L1^™^ localisation and polar lipids, we also investigated the distribution of lipids in lipid droplets using *Drosophila* fat body tissue using Raman spectroscopy. *Drosophila* fat body tissue was chosen as it is known that under normal feeding conditions, ReZolve-L1^™^ is detected throughout the lipid droplets of fat body cells from third instar larvae, 4 hours prior to puparium formation (-4 h PF); as visualised by confocal microscopy (Fig 3A)[41]. Therefore, to confirm the distribution of lipids (heterogeneous vs homogenous), especially in the core of lipid droplets, Raman spectra were collected from fed -4 h PF larvae. Tissue was not stained with ReZolve-L1^™^ to prevent signal overlap in key cellular areas and to better ascertain the composition of lipid droplets. The tissue was fixed in paraformaldehyde to ensure consistency between imaging experiments; from experience with this tissue, this fixation method preserves ultra-structure [58]. The location of lipid droplets were mapped by integrating the area under the **ν**(C = C) band from 1625–1675 cm^-1^ (Fig 3B), as the bands in this region have a contribution from both polar and neutral lipid species [57]. A number of spectra were extracted from the lipid droplet core (for representative spectra see S3 Fig), and spectral variations were observed in regions attributed to the **ν**(C-C) and **ν**((P-O) region at 1050-1150cm^-1^, the **δ**(C-H) region at 1400–1500 cm^-1^ and the **ν**(C = O) region at 1720-1780cm^-1^ [57], which indicated that a range of lipids were contributing to the spectra and are present in the lipid droplet core.

Further interrogation of the second derivative of the spectra taken from the lipid droplet revealed the presence of a band at ~ 1096cm^-1^ attributed to the **ν**(P-O), which is unique to phospholipids, such as phosphatidylcholine, phosphatidylethanolamine and sphingomyelin [57] (Fig 3C). Overlaying the Raman map of the **ν**(P-O) band (from the second derivative) with the **ν**(C = O) band, for the detection of triglycerides and sterol esters [57] it was possible to assess the relative location of the phospholipids (Fig 3D). The phospholipids were found to predominantly reside around the lipid droplet edges, but were also detected in the lipid droplet core. Hence, supporting the premise that lipid droplets are quite heterogeneous with respect to the distribution of polar lipids, which may account for the staining of ReZolve-L1^™^ in the lumen of *Drosophila* fat body lipid droplets.

The accumulation of data from both the *ex vivo* models (lipid overlay experiments and liposome analysis) combined with Raman spectroscopy of both a 3T3 adipocyte cell line and *Drosophila* tissue provide evidence for the localisation of ReZolve-L1^™^ in cells with polar lipids. With the Raman data suggesting that lipid droplets are more heterogeneous in lipid composition, which may account for the dispersed staining of lipid droplets with ReZolve-L1^™^, rather than an interaction with non-polar lipids. However, it should be noted that this does not discount an interaction between ReZolve-L1^™^ and non-polar lipids (such as CEs and/or TAGs), but that the evidence suggests a preferential interaction with polar lipids. The authors suggest that the observed localisation patterns of ReZolve-L1^™^ is not due to an interaction with one ‘specific’ lipid, but more closely aligned with the polar lipid families and localisation pattern may in fact be the result of a particular combination of lipid(s).

### Autophagy induction by complete starvation of *Drosophila* larvae lead to ReZolve-L1^™^ detection in autophagic compartments

Autophagosomes are cellular compartments that are involved in cell degradation and like lipid droplets can accumulate lipids from a range of cellular components [59, 60]. Probes that are described for use as lipid droplet markers [61] have also been shown to detect autophagosomes in some cell types [60]. Therefore, to investigate if ReZolve-L1^™^ could also detect autophagosomes, *Drosophila* fat bodies expressing the autophagic marker Atg8-GFP were stained with ReZolve-L1^™^ following exposure of the larvae to different autophagy inducing starvation conditions. Staining with Oil Red O was performed in parallel to allow for a comparison between a purely non-polar lipid dye and ReZolve-L1^™^; and to provide further evidence of the greater affinity for polar lipids.

**Fig 4:**
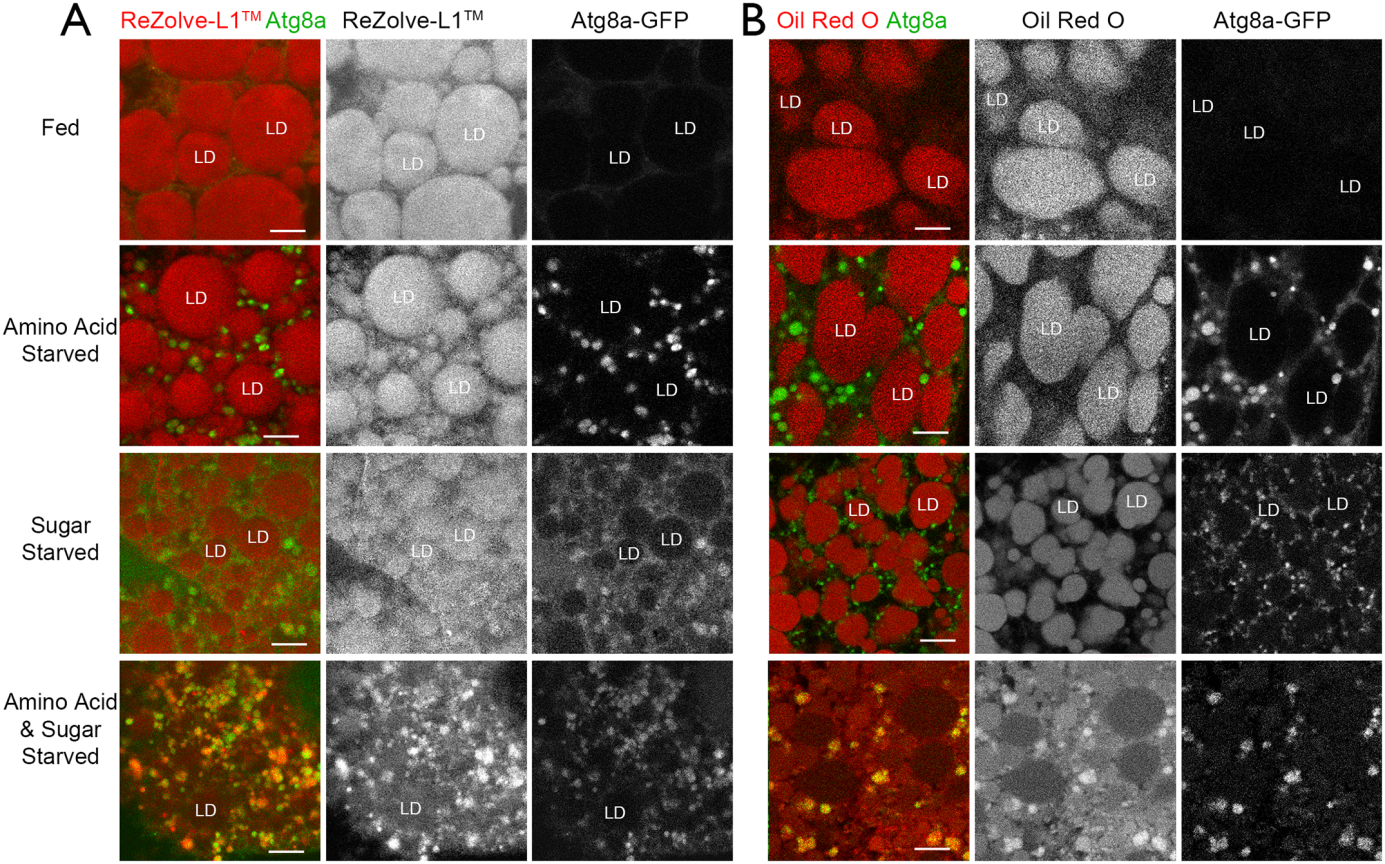
ReZolve-L1^™^ is detected in lipid droplets in fed larvae, but co-locates with Atg8aGFP autophagic compartments upon combined amino acid and sugar starvation. (**A**) Confocal micrographs of *Drosophila* fat body tissue from larvae at -4 h PF, stained with ReZolve-L1 (red) and expressing the autophagic marker *Atg8a-GFP* (green). Tissue was explanted from fed larvae or larvae exposed to 4 hours of amino acid, sugar or amino acid and sugar starvation. (**B**) Confocal micrographs of *Drosophila* fat body tissue from larvae at -4 h PF, stained with Oil Red O and expressing the autophagic marker *Atg8a-GFP*. Tissue was explanted from fed larvae or larvae exposed to 4 hours of amino acid, sugar or amino acid and sugar starvation. LD indicates lipid droplets. Scale bars = 10 μm.

Under normal feeding conditions, there were few Atg8a-GFP autophagic compartments and ReZolve-L1^™^ was detected mainly in lipid droplets (Fig 4A, Fed) as was Oil Red O (see S4 Fig). Larvae exposed to either amino acid starvation, sugar starvation or both amino acid and sugar starvation had significantly increased numbers of Atg8a-GFP autophagic compartments in the fat body tissue (*P*<0.05; Fig 4A and 4B), which confirmed the up regulation of autophagy under these different starvation conditions. While the lipid droplets in the fat body tissues from amino acid starved larvae were detected by ReZolve-L1^™^, there was minimal ReZolve-L1^™^ staining of Atg8a-GFP autophagic compartments (Fig 4A; amino acid starved). Oil Red O staining following amino acid starvation was also unchanged from normal feeding conditions (Fig 4B; amino acid starved). In the sugar starved larvae there were some changes in the ReZolve-L1^™^ staining of lipid droplets and some evidence of ReZolve-L1^™^ colocation with Atg8a-GFP autophagic compartments (Fig 4A; sugar starved). In contrast, the tissue staining of Oil Red O did not alter between normal conditions, amino acid starved or sugar starved (Fig 4B; sugar starved). Thus, while these conditions of nutrient restriction (amino acid or sugar starved) induced the formation of Atg8a-GFP autophagic compartments (as indicated by the expression of the green autophagic marker *Atg8a-GFP*), these compartments had minimal to no Oil Red O staining (Fig 4B). The lack of observable change in the Oil Red O staining pattern suggested that the overall lipophilicity of the lipid droplets in the *Drosophila* did not alter under these particular starvation conditions, and neutral lipids were not incorporated into autophagosomes. Notably, the lipid droplets detected by Oil Red O under these conditions had an elongated appearance when compared to those detected by ReZolve-L1^™^, which was most likely the result of lipid droplet fusion in the former, caused by cell fixation [9, 35, 36]. The change in ReZolve-L1^™^ staining pattern under conditions of sugar starvation, but no change in Oil Red O, suggested that the lipid target of ReZolve-L1^™^ differs from that of Oil Red O.

Interestingly, in the fat body tissue of larvae exposed to both amino acid and sugar starvation there was reduced ReZolve-L1^™^ staining in the lipid droplets and ReZolve-L1^™^ co-located with 90 ± 11% of Atg8a-GFP autophagic compartments (Fig 4A; amino acid & sugar starved). Furthermore, following amino acid and sugar starvation there appeared to be a reduction in the size of lipid droplets stained by Oil Red O and there was co-location of Oil Red O with Atg8a-GFP autophagic compartments (Fig 4B; amino acid & sugar starved). This suggested that under conditions of intense starvation (amino acid and sugar starved) that there was a significant change in the lipid composition of the lipid droplet and the Atg8a-GFP positive compartments. Hence, both dyes were found to co-localise with the autophagic marker positive compartments.

### ReZolve-L1^™^ staining in response to altered autophagic progression

**Fig 5:**
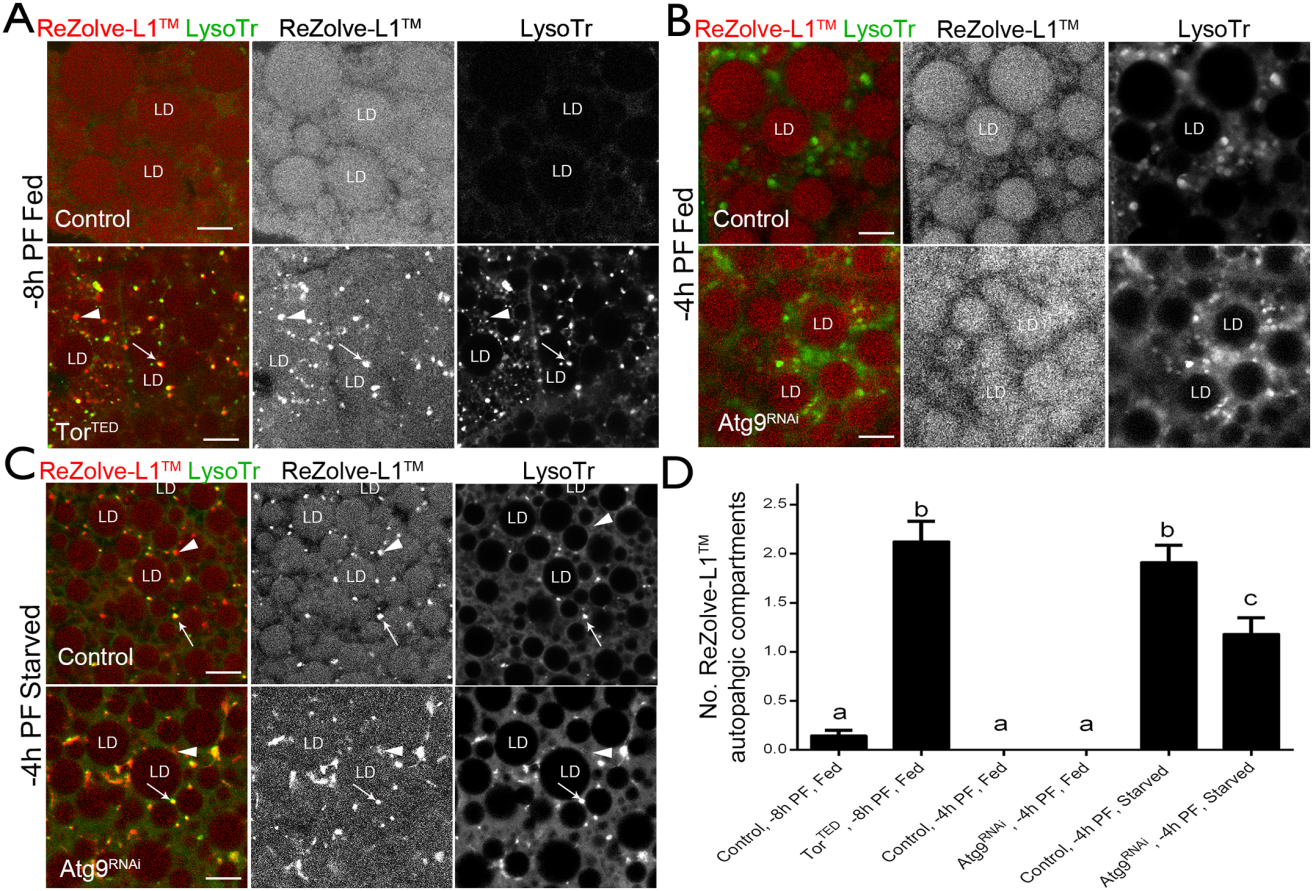
ReZolve-L1^™^ responds to altered autophagy. Confocal micrographs of *Drosophila* fat body tissue stained with the ReZolve-L1^™^ (red) or LysoTracker^®^ Green to detect autophagic activity. (**A**) Representative images from control or *Tor*^*TED*^ larvae at -8 h PF, fed on standard feeding media (-8 h PF Fed). (**B**) Representative images from control or Atg9^RNAi^ larvae at -4 h PF, fed on standard feeding media (-4 h PF Fed) (**C**) or exposed to 4 hours of amino acid and sugar starvation (-4 h PF Starved). LD indicates lipid droplets; arrows indicate co-location between ReZolve-L1^™^ and LysoTracker^®^ Green; arrow heads indicate ReZolve-L1^™^ only compartments. Scale = 10 μm. (**D**) Histogram showing the number of ReZolve-L1^™^ autophagic compartments per 100 μm^2^ of cell area. Mean ± s.e.m. presented for control or *Tor*^*TED*^ larvae at -8 h PF, fed on standard feeding medium (-8 h PF, Fed) and control or Atg9^RNAi^ larvae at -4 h PF, fed on standard media (-4 h PF, Fed) or exposed to 4 hours of amino acid and sugar starvation (-4 h PF, Starved). Groups are compared by ANOVA with Tukey post-hoc test, different letters indicates significance difference between means, P < 0.05.

The detection of autophagic compartments by ReZolve-L1^™^ was also assessed in *Drosophila* larval fat body during constitutive autophagy activation, induced by the expression of dominant negative *Tor* (*Tor*^*TED*^) [62, 63]. Fat body tissue was collected for imaging from third instar larvae at -8 h PF; as the expression of *Tor*^*TED*^ reduced the fat body volume in larvae at later developmental time points, which prevented the analysis. *Tor*^*TED*^ expression increased the number of autophagic compartments detected by LysoTracker^®^ Green in larval fat body tissue [50], when compared to control larval tissue (Fig 5A). The majority of the LysoTracker^®^ Green compartments detected in response to *Tor*^*TED*^ expression were co-stained by ReZolve-L1^™^, but there were some compartments that appeared to contain either only LysoTracker^®^ Green or only ReZolve-L1^™^ (Fig 5A). In contrast, the control tissues at -8 h PF had a limited number of LysoTracker^®^ Green compartments, and ReZolve-L1^™^ detected a minimal number of autophagic compartments (Fig 5D) and was primarily localised to the lipid droplets (Fig 5A). This again suggested that ReZolve-L1^™^ may be capable of detecting changes in compartments related to autophagy.

To further investigate the localisation of ReZolve-L1^™^ to autophagic compartments, autophagosome formation was disrupted by RNAi silencing of the core autophagy protein Atg9 [58, 64–66]. Control wild type larvae and larvae expressing *Atg9*^*RNAi*^, under the control of the fat body specific driver CG-GAL4, were either fed on standard media, or amino acid and sugar starved for four hours. In the control and Atg9^RNAi^ fed larvae ReZolve-L1^™^ was detected in lipid droplets and was not detected in LysoTracker^®^ Green compartments (Fig 5B). In control larvae responding to amino acid and sugar starvation, there was a significant increase in the number of autophagic compartments that were positive for ReZolve-L1^™^ and LysoTracker^®^ Green, when compared to fed larvae (Fig 5D), and the amount of ReZolve-L1^™^ staining in lipid droplets was reduced (Fig 5B and 5C). This was consistent with previous results from fat body tissue of both amino acid and sugar starved larvae. For Atg9^RNAi^ larvae that were amino acid and sugar starved, ReZolve-L1^™^ was detected in the core of the lipid droplets, however it did not clearly define these lipid droplets (Fig 5C). Although co-location between ReZolve-L1^™^ and LysoTracker^®^ Green still occurred in these starved Atg9^RNAi^ larvae (Fig 5C), there was a significant reduction in the number of these compartments in Atg9^RNAi^ larvae (Fig 5D). In Atg9^RNAi^ larvae, the LysoTracker^®^ Green compartments appeared as punctate structures that were similar to those seen in controls; however, ReZolve-L1^™^ did not appear as distinct puncta, but was detected in elongated structures distributed to regions near the periphery of the lipid droplets (Fig 5C). These results indicated that ReZolve-L1^™^ was responsive to changes in autophagy induced by genetic modulation. Furthermore ReZolve-L1^™^ detected changes in the number of autophagosomes formed in response to complete starvation, as well as morphological changes that could not be detected with other lipid markers.

### ReZolve-L1^™^ was detected in autophagic compartments in *Drosophila* fat body tissue during metamorphosis

**Fig 6:**
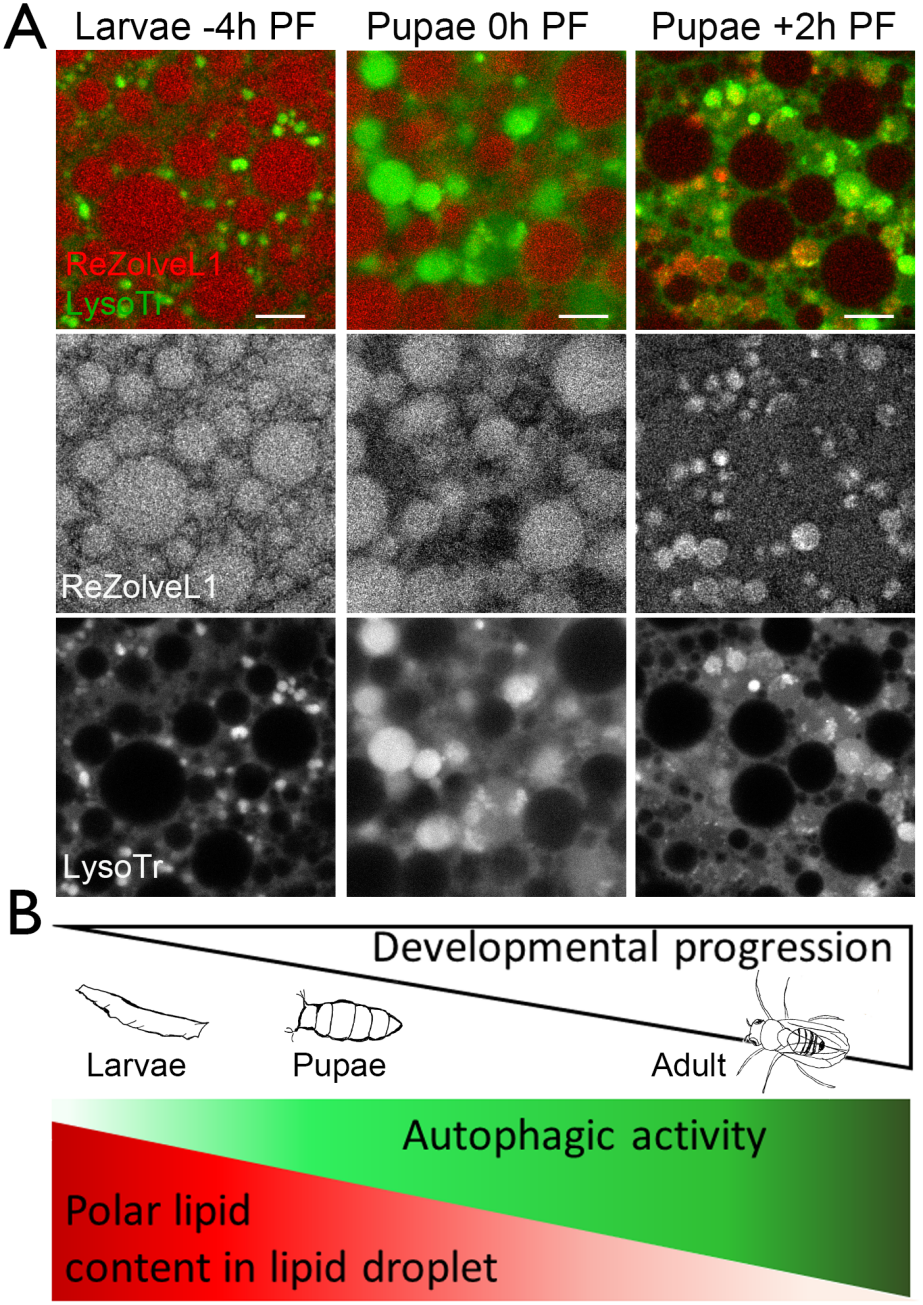
ReZolve-L1^™^ detects autophagic compartments in *Drosophila* fat body tissue during metamorphosis. (**A**) Confocal micrographs of fat body cells explanted from wild type *Drosophila* larvae and pupae, at either -4 h PF, 0 h PF, or +2 h PF and stained with ReZolve-L1^™^ (red) and LysoTracker^®^ Green. Scale bars = 10 μm. (**B**) Schematic of the relationship between *Drosophila* developmental progression, autophagic activity and polar lipid content in the lipid droplets of fat body tissue.

During *Drosophila* metamorphosis from larvae to adulthood there is increased autophagic activity [67, 68], which coincides with lipid mobilisation from lipid droplets that in turn supports the developmental process [69]. ReZolve-L1^™^ was used to investigate changes in lipid droplets and autophagic compartments in fat body tissue during the early stages of metamorphosis (-4 h PF, 0 h PF, +2 h PF). At -4 h PF ReZolve-L1^™^ was detected primarily in lipid droplets and not in LysoTracker^®^ Green compartments (Fig 6A). At 0 h PF the ReZolve-L1^™^ was still predominately in the lipid droplets, but the LysoTracker^®^ Green compartments were enlarged (when compared to those at -4 h PF) and these compartments had some ReZolve-L1^™^ staining (Fig 6A). At +2 h PF, the LysoTracker^®^ Green compartments had developed intraluminal vesicles that were co-stained with ReZolve-L1^™^ (Fig 6A). The lipid droplet staining detected by ReZolve-L1^™^ did not appear to change dramatically between the -4 h PF and 0 h PF developmental times points, but was reduced at + 2 h PF (Fig 6A). In contrast, the LysoTracker^®^ Green autophagic compartments had an increased size from -4 h PF to +2 h PF, and at +2 h PF displayed a morphology resembling either amphisomes or autolysosomes [58] (Fig 6A). ReZolve-L1^™^ co-located with Atg8a-GFP autophagic compartments at +2 h PF, which supported that these multivesicular structures were either amphisomes or autolysosomes (S4A Fig). In addition, at +2 h PF, these Atg8a-GFP autophagic compartments contained neutral lipids that were detected by Oil Red O staining (S4B Fig). These findings combined with our knowledge that ReZolve-L1^™^ has a stronger staining preference for polar lipids indicated that the polar lipid content in the lipid droplets may decrease during development. We suggest that this change in content may involve trafficking to autophagic compartments (graphic depiction Fig 6B).

## Discussion

Lipid droplets are most often described as cytosolic organelles consisting of a neutral lipid core, surrounded by a monolayer of phospholipids [11, 70]. Although the neutral lipid core contains primarily triglycerides and sterol esters, there is some evidence to suggest that polar lipids, such as cholesterol and phospholipids, can also reside in the lipid droplet core [71–74]. Biochemically, free cholesterol is able to partition into a neutral lipid phase in small amounts and in a biological system it is possible that this process may be further facilitated by proteins or other lipids [71, 72]. Electron microscopy of lipid droplets has demonstrated internal onion like layers of what appears to be phospholipids that are positive for the membrane associated protein caveolin, and the presence of phospholipids in lipid droplets was further conformed by mass spectrometry [70, 75]. The polar lipid sphingomyelin has also been shown to co-localise with neutral lipids stained by Nile-Red within fibroblast lipid droplets [73]. Raman mapping provided further evidence for the presence of phospholipids in the core of lipid droplets from *Drosophila* fat body tissues. In these tissues ReZolve-L1^™^ was also localised to lipid droplets and was detected throughout the organelle. Although the mode of action of ReZolve-L1^™^ requires further analysis, lipid overlay experiments, liposome affinity experiments and Raman mapping of stained adipocytes showed positive correlations between ReZolve-L1^™^ and polar lipids to a much greater extent than neutral lipids. Although we cannot rule out that the lipophilic nature of ReZolve-L1^™^ drives the localisation of this molecular probe to the lipid droplet core, it is speculated that ReZolve-L1^™^ is localising with and hence detecting polar lipids in both the outer membrane and the core of lipid droplets. This hypothesis is supported by Fourier transform infrared microspectroscopy, which demonstrated a correlation between the location of polar lipid species such as phosphatidylethanolamine and sphingomyelin and ReZolve-L1^™^ in 3T3-L1 adipocytes [76]. Interestingly, despite the apparent affinity for polar lipids, ReZolve-L1^™^ was most often detected in lipid droplets and was rarely associated with other cellular membrane structures where polar lipids are known to be abundant. This lack of other cell membrane staining may indicate that polar lipid packing in these membranes prevents ReZolve-L1^™^ interaction or that the interaction site is masked, or alternatively that ReZolve-L1^™^ only detects certain combinations of polar lipids.

Autophagy has a pivotal role in lipid localisation, for example, to orchestrate the vesicular traffic and secretion of triglyceride rich lipoproteins, as well as to mediate the transient storage of lipids in lipid droplets [20, 76, 77]. Autophagosomes can also recruit lipids for membrane expansion [78] and have a specific role in lipid degradation, which subsequently fuel the energy generating pathways involved in mitochondrial β-oxidation [79]. Consequently, the dynamic relationship that is emerging between lipids, lipid droplets and autophagy appears to be central in the key cell biological processes of energy sensing and metabolism [79]. ReZolve-L1^™^ was detected in autophagic compartments in response to certain starvation conditions, the inactivation of the target of Rapamycin (*Tor*^*TED*^), or developmental cues. The ReZolve-L1^™^ staining of lipid droplets was also altered in response to these cues, and these changes in distribution may involve the trafficking of polar lipids during interactions between lipid droplets and autophagosomes.

The lipids detected by ReZolveL1^™^ appeared to be directed to autophagosomes as a selective response to certain autophagic cues, rather than as a general response to autophagy induction. Under conditions of partial nutrient restriction by amino acid starvation, autophagosomes were formed, but ReZolve-L1^™^ had little interaction with these autophagic compartments. This would suggest that ReZolve-L1^™^ did not detect the polar lipids acquired from all cellular organelles that might be degraded under these conditions. ReZolve-L1^™^ did, however, co-locate with Atg8-GFP autophagic compartments following combined amino acid and sugar starvation, which indicated that it was recognised a specific event during autophagy. Amino acid and sugar starvation did induce lipid droplet degradation via autophagy, as shown by Oil Red O co-localisation with autophagosomes. While it is commonly accepted that neutral lipids are transferred to autophagic compartments in response to autophagy induction, by either combined amino acid and sugar starvation or the inhibition of Tor [15, 62, 63], other lipids and proteins are also known to be transferred under these conditions [11, 80]. Thus, it is possible that ReZolve-L1^™^ detects polar lipid sequestration from lipid droplets by autophagosomes under these conditions. The down regulation of autophagy by genetic inhibition is known to reduce lipid transfer from lipid droplets to autophagosomes [15, 81], and this was consistent with a decrease in ReZolve-L1^™^ detection in autophagosomes following autophagic reduction by Atg9^RNAi^ knock down. Atg9^RNAi^ knock down did not completely abrogate autophagic progression [58], but caused the formation of disseminated ReZolve-L1^™^ positive structures near the periphery of the lipid droplets; which could be indicative of altered autophagosome formation and reduced incorporation of lipid into autophagosome membrane, a process in which Atg9 is suggested to be involved [82, 83]. The inclusion of lipid droplets into autophagosomes can occur together with the inclusion of lipid membranes, for example from other organelles and the plasma membrane, resulting in increased levels of cholesterol, sphingolipids and GM1 in autophagosomes [84]. It is unclear in *Drosophila* fat body, if lipid membrane content in autophagosomes is increased under certain conditions, however this may be an alternative explanation for the detection of ReZolve-L1^™^ in autophagic compartments.

Interestingly, ReZolve-L1^™^ had an altered staining pattern upon sugar starvation alone, but unlike exposure to combined amino acid and sugar starvation, similar changes were not observed in Oil Red O staining. This difference in staining patterns supports the interaction of ReZolve-L1^™^ with lipids that are distinct from the neutral lipids detected by Oil Red O. Furthermore, this suggested that the polar lipids detected by ReZolve-L1^™^ have increased association with autophagic compartments during the energy depravation induced by sugar starvation. The induction of autophagy by most signalling pathways converge at TOR but in contrast, energy depletion can lead to the direct activation of autophagy via interactions between energy sensing AMPK and the autophagy initiator Atg1/ULK1 [85]. Following sugar starvation ReZolve-L1^™^ staining was detected throughout the cells with some brighter puncta seen localising with autophagic structures, unlike the staining observed following combined amino acid and sugar starvation, which was highly concentrated in autophagic compartments. This suggested a change in polar lipid distribution under these conditions, which may be associated with either an alternative signalling pathway being activated or a specific event in autophagosome maturation.

During *Drosophila* development, autophagy is important for the degradation and remodelling of larval tissue, including the fat body [68, 86]. The fat body lipid droplets are the major site of lipid storage in *Drosophila*, and these lipids can be mobilised to provide energy or used as structural lipids for developing tissues during metamorphosis [45, 87]. In *Drosophila*, the mobilisation of lipids during metamorphosis is in part facilitated by lipases, such as the lipid droplet associated Brummer and lysosomal acid lipase-1 [87–90]. The involvement of lysosomal associated lipases [87, 89] and the identification of autophagy as a route for lipid delivery to the lysosome in other systems [15] has led to speculation that autophagy has a critical function during *Drosophila* metamorphosis [86, 91]. In *Drosophila* fat body, at the later developmental stage of +2 h PF, ReZolve-L1^™^ staining was detected in autophagic compartments that were co-stained by LysoTracker^®^Green and Atg8a-GFP. Neutral lipids were also detected in these autophagic compartments at +2 h PF, which provided the first evidence of autophagy as a route for lipid delivery to the lysosome during metamorphosis. The detection of both neutral and polar lipids in the autophagosomes at the later developmental time point may also indicate that bulk degradation of lipid droplets is occurring at this stage.

There is a paucity of live cell imaging molecular probes available, to allow the specific investigation of lipids and to support the wider field of lipid metabolism. ReZolve-L1^™^ appears to be able to identify the transfer of polar lipids from lipid droplets to autophagic compartments under conditions of high energy demand and during developmental autophagy. By reporting on this dynamic balance between lipid droplets and autophagic compartments during different energy requirements, ReZolve-L1^™^ has the potential to define new aspects of cellular lipid metabolism. ReZolve-L1^™^ overcomes many of the critical limitations for conventional molecular probes, including photobleaching, extended sample preparation, and the need for cell fixation, which could enable live cell imaging of specific metabolic events.

The bidirectional interaction of autophagy and lipid metabolism has important implications for disease pathophysiology and there is a defined need to develop new imaging technology for these disease processes [18]. Consequently, ReZolve-L1^™^ could be used to visualise the pathology in for example: the altered lipid metabolism associated with fatty liver disease; the increased autophagy and altered degradation of lipids observed in Alzheimer’s disease; the altered cholesterol deposition and plaque formation in cardiovascular disease; or the altered balance between lipid metabolism and autophagy in cancer. Therefore ReZolve-L1^™^ may provide a promising new tool for the live cell imaging of polar lipids and autophagy that has practical applications for both cell biology research and clinical diagnosis/prognosis.

## Material and Methods

### Interaction of ReZolve-L1^™^ with lipids

To determine the binding properties of the rhenium molecular probe, a lipid overlay was performed. 50 μM of each lipid was loaded on to a PVDF membrane (Perkin Elmer, USA). Ten lipids were analysed including; sphingomyelin (Cat # S0756, Sigma Aldrich, USA), sphingosine (Cat # S7049; Sigma Aldrich, USA), ʟ-α-phosphatidylethanolamine (Cat# P7943, Sigma Aldrich, USA), monosialodanglioside (GM1; Cat # G7641, Sigma Aldrich, USA), oleoly-ʟ-α-lysophosphatidic acid (Cat # L7260, Sigma Aldrich, USA), palmitic acid (Cat # P5917, Sigma Aldrich, USA), cholesterol (Cat # CO01800100; Scharlau, German), ceramide (Cat # C2137; Sigma Aldrich, USA), triacylglycerol mix C2-C10 (Cat # 17810, Sigma Aldrich, USA) and cholesteryl acetate (Cat # 151114; Sigma Aldrich, USA). Lipids were dissolved in ethanol or *iso*-propanol for loading depending on solubility. Following lipid loading, the PVDF membranes were washed for 10 minutes in cold 10% ethanol, methanol or PBS, before they were incubated with 10μM of rhenium molecular probe in PBS for 1h. The neutral rhenium(I) species, ReZolve-L1^™^, was prepared according to a previously published procedure [41]. PVDF membranes were then washed four times for 10 minutes in cold 10% ethanol, methanol or PBS prior to imaging. Each protocol was carried out in triplicate. Imaging was performed on an Image Quant Las 4000 (GE, Sweden) luminescent image analyser, with the rhenium molecular probe excitated at 460nm using an epi-blue light source, and detected using an 575 nm ethidium bromide filter.

### Liposome preparation

1,2-dimyristoyl-*sn*-glycero-3-phosphocholine (DMPC; Cat # 850345, Avanti Polar Lipid, Inc. Alabaster, USA) and cholesterol (Cat # CO01800100; Scharlau, German), were dissolved in 2:1 chloroform:methanol at 20 mg/mL. Lipid mixtures were formed by evaporating liquid 666 μL DMPC alone or a mixture containing 443 μL of DMPC and 32 μL of cholesterol (8:1 molar ratio). The lipid mixture was then resuspended in 1 mL PBS and sonicated for 40 minutes. Following sonication liposomes were passed through an extruder with a 100 nm membrane 15 times. Liposome formation was confirmed using a Malvern Zetasizer DLS system. Mixtures were run through a Sephadex G-25 column to ensure no free lipid was present in solution. Fraction containing particles of approximately 100 nm (ascertained by DLS) were incubated with ReZolve-L1^™^ for 40 minutes. Solutions were then passed through a Sephadex G-25 column to remove unbound stain and fraction containing vesicles of approximately 100 nm were tested for fluorescence’s by flow cytometry and fluorometry.

For analysis of vesicles by flow cytometry an Amnis ImageStreamX Flow Cytometer mkII (EMD Millipore) was used. Vesicles were excited with 405 nm laser (120 mW) and fluorescent data between 560–595 nm were collected. In parallel, data for forward scatter (FSC) and side scatter (SSC) light (785 nm laser, 5.6 mW) were collected. Core diameter of the flow was 10 μm and flow speed was 66 mm/sec. Calibration beads (2 μm in diameter) were run in parallel to the samples. For each sample, images for 1.5–5.0x10^4^ events were collected using 40x objective and camera with pixel size of 0.5 μm. Vesicles were identified by their low SSC signal and high fluorescence (405/560-595 nm) values; the accuracy of this separation was proven by viewing the images of single events.

For analysis of vesicles by fluorescence a Varian Cary Eclipse spectrophotometer was used. Vesicles were excited at 256 nm and emission was collected from 350–700 nm. Vesicles were tested for fluorescence before and after incubation with ReZolve-L1^™^.

### Cell culture and fly stocks

Mammalian 3T3 adipocytes were obtained from culturing and differentiated the 3T3 pre-adipocyte fibroblast on calcium fluoride slides for Raman microscopy, as previously described [92] for a minimum of three weeks before experimentation [37]. All fly stocks were maintained in standard medium at 25°C. The yeast *GAL4-UAS* system was used for targeted gene expression [93]. Fat-body specific expression of transgenes from the UAS were driven by *CG-GAL4* [94]. Transgenic *UAS-Atg9*^*RNAi*^ stocks (#34901) and *UAS-Tor*^*TED*^ stocks (#7013) were obtained from the Bloomington *Drosophila* Stock Centre (Indiana University, Indiana, USA). *Atg8a-GFP* was driven by an endogenous promoter [95] and was kindly provided by Dr Erik Baehrecke (University of Massachusetts Medical School, Massachusetts, USA).

### Developmental staging

Bromophenol-blue was added to the fly media and used to define different developmental time points [96]. Third instar larvae at minus eight hours of puparium formation (-8 h PF) had a blue gut and were observed wandering in and out of the media, whereas at minus four hours (-4 h PF) the guts had little or no blue dye and the larvae were only observed out of the media. Newly formed white pupae (0 h PF), were isolated onto moist filter paper and incubated at 25°C for two hours (+2 h PF).

### Nutrient deprivation

Third instar larvae at -8 h puparium formation (PF) were removed from standard media and placed on filter paper either soaked in 5% (w/v) sucrose (for amino acid deprivation), or 5% (w/v) tryptone pancreatic digest of casein (BD Bioscience USA; for sugar deprivation), or soaked in water (for amino acid and sugar starvation). Larvae were incubated at 25°C for 4–5 hours prior to tissue isolation and imaging.

### Tissue isolation and staining

The neutral rhenium(I) molecular probe ReZolve-L1^™^ was prepared according to a previously published procedure [41]. ReZolve-L1^™^ was dissolved in DMSO to prepare a 10 mM stock solution, which was diluted in PBS to 10 μM for tissue staining. For Raman confocal microscopy, 3T3 cells were fixed using cold methanol and air dried [37] before being incubated with ReZolve-L1^™^ overnight. Fat body tissue was dissected from *Drosophila* larvae or pupae into PBS. For Raman confocal microscopy, the tissue was fixed in 4% paraformaldehyde (Sigma Aldrich, St Louise, USA) ready for mapping. For live confocal and two photon microscopy, the fat body tissue was incubated with ReZolve-L1^™^ for 15 minutes at room temperature and washed in PBS for 30 seconds. Tissues were counterstained for acidic compartments with LysoTracker^®^ Green (prepared according to manufacturer instructions; Invitrogen, USA) for 2 minutes at room temperature. Tissues were then mounted in carbomer-940 (Snowdrift farm, Tucson, USA) based optical coupling gel to prevent dehydration prior to imaging; see protocol in [97]. For Oil Red O staining, fat body tissue was fixed in 2% (v/v) paraformaldehyde for 20 minutes at room temperature. Tissues were then washed three times in PBS for ten minutes before incubation in 1/100 Oil Red O for 30 minutes and then washed in PBS before mounting in 80% glycerol.

### Raman mapping, confocal imaging and analysis

Raman spectra were collected using a Renishaw Raman inVia Reflex Microscope (Renishaw plc., Wotton-under-Edge, UK), equipped with an air-cooled charge-coupled device (CCD) cameras for StreamLine^™^ HR mapping. The spectrometer was fitted with holographic notch filters and 2400 mm/line (NIR) grating. The attached microscope was a Leica DMLM and was equipped with three objectives (×50/0.75 NA, ×20/0.40 NA, ×5/0.12 NA) and a trinocular viewer that accommodates a video camera allowing direct viewing of the sample. Sample excitation was achieved using a Modu-Laser (Utah, USA) emitting at 785 nm. Calibration of the wavenumber axis is achieved by recording the Raman spectrum of silicon (1 accumulation, 10 seconds) for both static and extended modes. If necessary an offset correction is performed to ensure that the position of the silicon band is at 520.50 ± 0.10 cm^-1^. Spectra were not corrected for instrument response. The spectrometer was controlled by PC with instrument control software (Renishaw WiRE^™^, Version 4.2). The Raman maps were acquired using a step size of 2 μm for both *x* and *y* axes over the spectral range of 700–1850 cm^-1^.

Cosmic rays were removed from the spectral data using a nearest neighbour cosmic ray removal method and noise filtering was applied using the Renishaw WiRE 4.2 software. The false colour maps shown generated from the data using the direct analysis approach by calculating the area under bands of interest using the signal-to-baseline mode.

A Ziess LSM710 META NLO confocal microscope (Zeiss, Germany), supplemented with a two-photon Mai-Tai^®^ (tunable Ti:Sapphire femtosecond pulse laser, 710–920 nm, Spectra-Physics, Australia) was used for live imaging. The images were acquired using a Plan- APOCHROMAT 63X/ NA1.4 oil immersion objective. ReZolve-L1^™^ was excited at 830 nm using the two-photon pulse laser and detect at 600–654 nm. LysoTracker^®^ Green was imaged by excitation at 488 nm and detected at 497–558 nm. Atg8a-GFP was imaged with ReZolve-L1^™^ by excitation at 830 nm (to excite both fluorophores) and resolved using spectral un-mixing using the Zen software package (based on spectral Figprinting of the two components). A spectral profile for ReZolve-L1^™^ was acquired in lambda stack mode (830 nm excitation range, MBS 690+, spectral emission range 416–727 nm, with 9.7 nm intervals).

The ReZolve-L1^™^ images and co-location analysis involved at least five separate images from five independent biological replicates, and one representative image from each treatment was selected for presentation. For image quantification, two regions of interest (ROI) were selected at random, with an area of 2500 μm^2^ and the number of compartments stained with ReZolve-L1^™^ and LysoTracker^®^Green were counted to give a minimum of ten counts per treatment group. Comparison of means was performed by ANOVA with a Tukey post hoc tested in GraphPad Prism V.6.01 (USA).

## Supporting Information

S1 Fig Fluorometer of liposomes incubated with ReZolve-L1^™^. Histogram showing fluorescence intensity 560-595nm of DMPC vesicles (**A**) and DMPC and cholesterol vesicles (DMPC:cholesterol 8:1 molar ratio) (**B**) incubated with ReZolve-L1^™^ when excited at 405nm and representative fluorescence and transmitted lights images of vesicles by flow cytometry. (**C**) Fluoroscence spectra of liposomes alone (dashed grey line), ReZolve-L1^™^ alone (dotted grey line) and liposomes incubated with ReZolve-L1^™^ (black line; DMPC dashed line, DMPC and cholesterol solid line) when excited at 256nm.
(TIF)

S2 Fig Raman spectra of ReZolve-L1^™^ and ReZolve-L1^™^ stained 3T3 cells. (**A**) Structure and Raman spectrum of ReZolve-L1^™^ and (**B**) the second derivative of this spectrum. (**C**) Second derivatives of Raman spectra taken from a ReZolve-L1^™^ stained 3T3 adipocytes from a region of high (a) medium (b) and low (c) ReZolve-L1^™^ presence.
(TIF)

S3 Fig Raman spectra from *Drosophila* lipid droplet core. Representative Raman spectra from the lipid droplet core of -4 h PF larval fat body tissue. Corresponding second derivatives are presented above each of the five spectra. Important lipid regions are shaded and assigned above.
(TIF)

S4 Fig ReZolve-L1^™^ and Oil Red O co-locate with Atg8a-autopahgic compartments in *Drosophila* fat body tissue during metamorphosis. (**A**) Confocal micrographs of *Drosophila* fat body cells explanted from +2 h PF from pupae expressing Atg8a-GFP (green) and stained with ReZolve-L1^™^ (red). (**B**) Confocal micrographs of *Drosophila* fat body cells explanted from +2 h PF from pupae expressing Atg8a-GFP (green) and stained with Oil Red O (red). LD indicates lipid droplets arrows indicate co-location between ReZolve-L1 or Oil Red O and Atg8a-GFP autophagic compartments. Scale = 10 μm.
(TIF)

## References

[1] Liu H, Jia Q, Tettamanti G, Li S. Balancing crosstalk between 20-hydroxyecdysone-induced autophagy and caspase activity in the fat body during Drosophila larval-prepupal transition. Insect Biochem Mol Biol. 2013;43(11):1068–78. 10.1016/j.ibmb.2013.09.001 24036278

[2] Baenke F, Peck B, Miess H, Schulze A. Hooked on fat: the role of lipid synthesis in cancer metabolism and tumour development. Disease Models & Mechanisms. 2013;6(6):1353–63. 10.1242/dmm.011338 24203995 PMC3820259

[3] Haberkant P, Holthuis JCM. Fat & fabulous: Bifunctional lipids in the spotlight. Biochimica et Biophysica Acta (BBA)—Molecular and Cell Biology of Lipids. 2014;1841(8):1022–30. 10.1016/j.bbalip.2014.01.003. 24440797

[4] Dirkx R, Solimena M. Cholesterol-enriched membrane rafts and insulin secretion. Journal of Diabetes Investigation. 2012;3(4):339–46. 10.1111/j.2040-1124.2012.00200.x. WOS:000307961300002. 24843586 PMC4019251

[5] Lang T. SNARE proteins and 'membrane rafts'. J Physiol. 2007;585(Pt 3):693–8. 10.1113/jphysiol.2007.134346 17478530 PMC2375502

[6] Lingwood D, Simons K. Lipid Rafts As a Membrane-Organizing Principle. Science. 2010;327(5961):46–50. 10.1126/science.1174621. WOS:000273395400025. 20044567

[7] Chiantia S, London E. Sphingolipids and membrane domains: recent advances. Handbook of Experimental Pharmacology. 2013;(215):33–55. Epub 2013/04/13. 10.1007/978-3-7091-1368-4_2 . 23579448

[8] Hannich JT, Umebayashi K, Riezman H. Distribution and functions of sterols and sphingolipids. Perspect Biol. 2011;3(5). Epub 2011/04/02. 10.1101/cshperspect.a004762 ; PubMed Central PMCID: PMCPmc3101845. 21454248 PMC3101845

[9] Melo RC, D'Avila H, Wan HC, Bozza PT, Dvorak AM, Weller PF. Lipid bodies in inflammatory cells: structure, function, and current imaging techniques. J Histochem Cytochem. 2011;59(5):540–56. 10.1369/0022155411404073 21430261 PMC3201176

[10] Ruggles KV, Turkish A, Sturley SL. Making, baking, and breaking: the synthesis, storage, and hydrolysis of neutral lipids. Annual Review of Nutrition. 2013;33:413–51. 10.1146/annurev-nutr-071812-161254 . 23701589

[11] Thiam AR, Farese RV Jr, Walther TC. The biophysics and cell biology of lipid droplets. Nat Rev Mol Cell Biol. 2013;14(12):775–86. 10.1038/nrm3699 24220094 PMC4526153

[12] Zehmer JK, Huang Y, Peng G, Pu J, Anderson RG, Liu P. A role for lipid droplets in inter-membrane lipid traffic. Proteomics. 2009;9(4):914–21. 10.1002/pmic.200800584 19160396 PMC2676673

[13] Thiam AR, Farese RV, Walther TC. The biophysics and cell biology of lipid droplets. Nature Reviews Molecular Cell Biology. 2013;14(12):775–86. 10.1038/Nrm3699. WOS:000327542100011. 24220094 PMC4526153

[14] Ouimet M, Franklin V, Mak E, Liao X, Tabas I, Marcel YL. Autophagy regulates cholesterol efflux from macrophage foam cells via lysosomal acid lipase. Cell metabolism. 2011;13(6):655–67. 10.1016/j.cmet.2011.03.023 21641547 PMC3257518

[15] Singh R, Kaushik S, Wang Y, Xiang Y, Novak I, Komatsu M, et al Autophagy regulates lipid metabolism. Nature. 2009;458(7242):1131–5. 10.1038/nature07976 19339967 PMC2676208

[16] van Zutphen T, Todde V, de Boer R, Kreim M, Hofbauer HF, Wolinski H, et al Lipid droplet autophagy in the yeast Saccharomyces cerevisiae. Mol Biol Cell. 2014;25(2):290–301. 10.1091/mbc.E13-08-0448 24258026 PMC3890349

[17] Koga H, Kaushik S, Cuervo AM. Altered lipid content inhibits autophagic vesicular fusion. FASEB J. 2010;24(8):3052–65. 10.1096/fj.09-144519 20375270 PMC2909278

[18] Liu K, Czaja MJ. Regulation of lipid stores and metabolism by lipophagy. Cell Death Differ. 2013;20(1):3–11. 10.1038/cdd.2012.63 22595754 PMC3524634

[19] Alexaki A, Gupta SD, Majumder S, Kono M, Tuymetova G, Harmon JM, et al Autophagy regulates sphingolipid levels in the liver. Journal of lipid research. 2014;55(12):2521–31. Epub 2014/10/22. 10.1194/jlr.M051862 ; PubMed Central PMCID: PMCPmc4242445. 25332431 PMC4242445

[20] Ouimet M. Autophagy in obesity and atherosclerosis: Interrelationships between cholesterol homeostasis, lipoprotein metabolism and autophagy in macrophages and other systems. Biochim Biophys Acta. 2013;1831(6):1124–33. Epub 2013/04/03. 10.1016/j.bbalip.2013.03.007 . 23545567

[21] Zhou W, Hoffmann R, Franke A, Yang H, Kuhl H, Hanrath P. Intravascular ultrasound evaluating coronary stents for patients with coronary artery disease: compared old with new multilink stents. Chinese Medical Sciences Journal. 2002;17(2):95–100. Epub 2003/08/09. . 12906162

[22] Inokuchi-Shimizu S, Park EJ, Roh YS, Yang L, Zhang B, Song J, et al TAK1-mediated autophagy and fatty acid oxidation prevent hepatosteatosis and tumorigenesis. The Journal of clinical investigation. 2014;124(8):3566–78. 10.1172/JCI74068 24983318 PMC4109552

[23] Cai N, Zhao X, Jing Y, Sun K, Jiao S, Chen X, et al Autophagy protects against palmitate-induced apoptosis in hepatocytes. Cell & Bioscience. 2014;4:28 Epub 2014/06/07. 10.1186/2045-3701-4-28 ; PubMed Central PMCID: PMCPmc4045884. 24904743 PMC4045884

[24] Tan SH, Shui G, Zhou J, Li JJ, Bay BH, Wenk MR, et al Induction of autophagy by palmitic acid via protein kinase C-mediated signaling pathway independent of mTOR (mammalian target of rapamycin). The Journal of Biological Chemistry. 2012;287(18):14364–76. Epub 2012/03/13. 10.1074/jbc.M111.294157 ; PubMed Central PMCID: PMCPmc3340233. 22408252 PMC3340233

[25] Xin W, Zhao X, Liu L, Xu Y, Li Z, Chen L, et al Acetyl-CoA carboxylase 2 suppression rescues human proximal tubular cells from palmitic acid induced lipotoxicity via autophagy. Biochem Biophys Res Commun. 2015;463(3):364–9. Epub 2015/05/30. 10.1016/j.bbrc.2015.05.070 . 26022126

[26] Li S, Li J, Shen C, Zhang X, Sun S, Cho M, et al tert-Butylhydroquinone (tBHQ) protects hepatocytes against lipotoxicity via inducing autophagy independently of Nrf2 activation. Biochim Biophys Acta. 2014;1841(1):22–33. Epub 2013/09/24. 10.1016/j.bbalip.2013.09.004 ; PubMed Central PMCID: PMCPmc3884638. 24055888 PMC3884638

[27] Park M, Sabetski A, Kwan Chan Y, Turdi S, Sweeney G. Palmitate induces ER stress and autophagy in H9c2 cells: implications for apoptosis and adiponectin resistance. J Cell Physiol. 2015;230(3):630–9. Epub 2014/08/29. 10.1002/jcp.24781 . 25164368

[28] Schnell U, Dijk F, Sjollema KA, Giepmans BN. Immunolabeling artifacts and the need for live-cell imaging. Nature methods. 2012;9(2):152–8. Epub 2012/02/01. 10.1038/nmeth.1855 . 22290187

[29] Gocze PM, Freeman DA. Factors underlying the variability of lipid droplet fluorescence in MA-10 Leydig tumor cells. Cytometry. 1994;17(2):151–8. Epub 1994/10/01. 10.1002/cyto.990170207 . 7835165

[30] Greenspan P, Mayer EP, Fowler SD. Nile red: a selective fluorescent stain for intracellular lipid droplets. The Journal of Cell Biology. 1985;100(3):965–73. Epub 1985/03/01. ; PubMed Central PMCID: PMCPmc2113505. 3972906 10.1083/jcb.100.3.965 PMC2113505

[31] Andersson L, Boström P, Ericson J, Rutberg M, Magnusson B, Marchesan D, et al PLD1 and ERK2 regulate cytosolic lipid droplet formation. Journal of Cell Science. 2006;119(11):2246–57. 10.1242/jcs.02941 16723731

[32] Spangenburg EE, Pratt SJ, Wohlers LM, Lovering RM. Use of BODIPY (493/503) to visualize intramuscular lipid droplets in skeletal muscle. Journal of biomedicine & biotechnology. 2011;2011:598358 Epub 2011/10/01. 10.1155/2011/598358 21960738 PMC3180081

[33] Gimpl G. Cholesterol-protein interaction: methods and cholesterol reporter molecules. Sub-cell Biochemistry. 2010;51:1–45. Epub 2010/03/10. 10.1007/978-90-481-8622-8_1 . 20213539

[34] Hackett MJ, McQuillan JA, El-Assaad F, Aitken JB, Levina A, Cohen DD, et al Chemical alterations to murine brain tissue induced by formalin fixation: implications for biospectroscopic imaging and mapping studies of disease pathogenesis. The Analyst. 2011;136(14):2941–52. Epub 2011/06/02. 10.1039/c0an00269k . 21629894

[35] Fujimoto T, Parton RG. Not just fat: the structure and function of the lipid droplet. Cold Spring Harbor Perspectives in Biology. 2011;3(3). Epub 2011/03/23. 10.1101/cshperspect.a004838 21421923 PMC3039932

[36] Fukumoto S, Fujimoto T. Deformation of lipid droplets in fixed samples. Histochemistry and Cell Biology. 2002;118(5):423–8. Epub 2002/11/15. 10.1007/s00418-002-0462-7 . 12432454

[37] Carter EA, Rayner BS, McLeod AI, Wu LE, Marshall CP, Levina A, et al Silicon nitride as a versatile growth substrate for microspectroscopic imaging and mapping of individual cells. Mol Biosyst. 2010;6(7):1316–22. 10.1039/C001499K 20445927

[38] Spandl J, White DJ, Peychl J, Thiele C. Live cell multicolor imaging of lipid droplets with a new dye, LD540. Traffic. 2009;10(11):1579–84. 10.1111/j.1600-0854.2009.00980.x . 19765264

[39] Maxfield FR, Wüstner D. Chapter 17—Analysis of Cholesterol Trafficking with Fluorescent Probes In: Gilbert Di P, Markus RW, editors. Methods in cell biology. Volume 108: Academic Press; 2012 p. 367–93. 10.1016/B978-0-12-386487-1.00017-1 PMC3626500 22325611

[40] Arthur JR, Heinecke KA, Seyfried TN. Filipin recognizes both GM1 and cholesterol in GM1 gangliosidosis mouse brain. Journal of lipid research. 2011;52(7):1345–51. 10.1194/jlr.M012633 21508255 PMC3122916

[41] Bader CA, Brooks RD, Ng YS, Sorvina A, Werrett MV, Wright PJ, et al Modulation of the organelle specificity in Re (I) tetrazolato complexes leads to labeling of lipid droplets. Royal Society of Chemistry Advances. 2014;4(31):16345–51. 10.1039/C4ra00050a. ISI:000334681800063.

[42] Vaughan JG, Reid BL, Ramchandani S, Wright PJ, Muzzioli S, Skelton BW, et al The photochemistry of rhenium(i) tricarbonyl N-heterocyclic carbene complexes. Dalton Transactions. 2013;42(39):14100–14. 10.1039/c3dt51614h 23939232

[43] Carter EA, Tam KK, Armstrong RS, Lay PA. Vibrational spectroscopic mapping and imaging of tissues and cells. Biophysical Reviews. 2009;1(2):95–103. 10.1007/s12551-009-0012-9 28509988 PMC5418372

[44] Zirin J, Perrimon N. Drosophila as a model system to study autophagy. Seminars in Immunopathology. 2010;32(4):363–72. 10.1007/s00281-010-0223-y 20798940 PMC3562086

[45] Kuhnlein RP. Thematic review series: Lipid droplet synthesis and metabolism: from yeast to man. Lipid droplet-based storage fat metabolism in Drosophila. Journal of lipid research. 2012;53(8):1430–6. Epub 2012/05/09. 10.1194/jlr.R024299 ; PubMed Central PMCID: PMCPmc3540836. 22566574 PMC3540836

[46] Kuhnlein RP. The contribution of the Drosophila model to lipid droplet research. Prog Lipid Res. 2011;50(4):348–56. Epub 2011/05/31. 10.1016/j.plipres.2011.04.001 . 21620889

[47] Juhasz G, Neufeld T. Experimental control and characterization of autophagy in Drosophila. Methods Mol Biol. 2008;445:125–33. 10.1007/978-1-59745-157-4_8 18425447

[48] Neufeld TP. Genetic manipulation and monitoring of autophagy in *Dosophila* In: Daniel JK, editor. Methods in enzymology. Volume 451: Academic Press; 2008 p. 653–67. 10.1016/S0076-6879(08)03236-9 19185744

[49] Scott R, Schuldiner O, Neufeld T. Role and regulation of starvation-induced autophagy in the Drosophila fat body. Dev Cell. 2004;7(2):167–78. 10.1016/j.devcel.2004.07.009 15296714

[50] Neufeld TP. Genetic manipulation and monitoring of autophagy in Drosophila. Methods in enzymology. 2008;451:653–67. Epub 2009/02/03. 10.1016/S0076-6879(08)03236-9 . 19185744

[51] Greenspan P, Mayer EP, Fowler SD. Nile red: a selective fluorescent stain for intracellular lipid droplets. The Journal of cell biology. 1985;100(3):965–73. Epub 1985/03/01. ; PubMed Central PMCID: PMCPmc2113505. 3972906 10.1083/jcb.100.3.965 PMC2113505

[52] Rudolf M, Curcio CA. Esterified cholesterol is highly localized to Bruch's membrane, as revealed by lipid histochemistry in wholemounts of human choroid. Journal of Histochemistry & Cytochemistry. 2009;57(8):731–9. 10.1369/jhc.2009.953448. WOS:000268122000003. 19365091 PMC2713073

[53] Wang JD, Douville NJ, Takayama S, ElSayed M. Quantitative analysis of molecular absorption into PDMS microfluidic channels. Annals of biomedical engineering. 2012;40(9):1862–73. Epub 2012/04/10. 10.1007/s10439-012-0562-z . 22484830

[54] Sahoo SK, Labhasetwar V. Nanotech approaches to drug delivery and imaging. Drug Discovery Today. 2003;8(24):1112–20. 10.1016/S1359-6446(03)02903-9. 14678737

[55] Kirby C, Clarke J, Gregoriadis G. Effect of the cholesterol content of small unilamellar liposomes on their stability in vivo and in vitro. The Biochemical journal. 1980;186(2):591–8. Epub 1980/02/15. ; PubMed Central PMCID: PMCPmc1161612. 7378067 10.1042/bj1860591 PMC1161612

[56] Digel M, Ehehalt R, Füllekrug J. Lipid droplets lighting up: Insights from live microscopy. FEBS Lett. 2010;584(11):2168–75. 10.1016/j.febslet.2010.03.035. 10.1016/j.febslet.2010.03.035 20347811

[57] Czamara K, Majzner K, Pacia MZ, Kochan K, Kaczor A, Baranska M. Raman spectroscopy of lipids: a review. J Raman Spectrosc. 2015;46(1):4–20. 10.1002/jrs.4607. WOS:000348501500002.

[58] Bader CA, Shandala T, Ng YS, Johnson IR, Brooks DA. Atg9 is required for intraluminal vesicles in amphisomes and autolysosomes. Biology Open. 2015;4(11):1345–55. 10.1242/bio.013979 . 26353861 PMC4728360

[59] Singh R, Cuervo AM. Lipophagy: connecting autophagy and lipid metabolism. International journal of cell biology. 2012;e2012:282041. 10.1155/2012/282041 22536247 PMC3320019

[60] Niemann A, Baltes J, Elsasser HP. Fluorescence properties and staining behavior of monodansylpentane, a structural homologue of the lysosomotropic agent monodansylcadaverine. The journal of histochemistry and cytochemistry: official journal of the Histochemistry Society. 2001;49(2):177–85. . 11156686 10.1177/002215540104900205

[61] Yang HJ, Hsu CL, Yang JY, Yang WY. Monodansylpentane as a blue-fluorescent lipid-droplet marker for multi-color live-cell imaging. PLOS ONE. 2012;7(3):e32693 10.1371/journal.pone.0032693 22396789 PMC3291611

[62] Scott RC, Schuldiner O, Neufeld TP. Role and Regulation of Starvation-Induced Autophagy in the Drosophila Fat Body. Developmental Cell. 2004;7(2):167–78. 10.1016/j.devcel.2004.07.009 15296714

[63] Noda T, Ohsumi Y. Tor, a phosphatidylinositol kinase homologue, controls autophagy in yeast. The Journal of biological chemistry. 1998;273(7):3963–6. Epub 1998/03/28. . 9461583 10.1074/jbc.273.7.3963

[64] Low P, Varga A, Pircs K, Nagy P, Szatmari Z, Sass M, et al Impaired proteasomal degradation enhances autophagy via hypoxia signaling in Drosophila. BMC cell biology. 2013;14:29 Epub 2013/06/27. 10.1186/1471-2121-14-29 23800266 PMC3700814

[65] Tang HW, Liao HM, Peng WH, Lin HR, Chen CH, Chen GC. Atg9 interacts with dTRAF2/TRAF6 to regulate oxidative stress-induced JNK activation and autophagy induction. Developmental Cell. 2013;27(5):489–503. Epub 2013/11/26. 10.1016/j.devcel.2013.10.017 . 24268699

[66] Nagy P, Hegedus K, Pircs K, Varga A, Juhasz G. Different effects of Atg2 and Atg18 mutations on Atg8a and Atg9 trafficking during starvation in Drosophila. FEBS Letters. 2014;588(3):408–13. Epub 2014/01/01. 10.1016/j.febslet.2013.12.012 24374083 PMC3928829

[67] Butterworth FM, Emerson L, Rasch EM. Maturation and degeneration of the fat body in the Drosophila larva and pupa as revealed by morphometric analysis. Tissue & Cell. 1988;20(2):255–68. Epub 1988/01/01. . 3136556 10.1016/0040-8166(88)90047-x

[68] Rusten T, Lindmo K, Juhasz G, Sass M, Seglen P, Brech A, et al Programmed autophagy in the Drosophila fat body is induced by ecdysone through regulation of the PI3K pathway. Developmental Cell. 2004;7(2):179–92. 10.1016/j.devcel.2004.07.005 15296715

[69] Aguila JR, Suszko J, Gibbs AG, Hoshizaki DK. The role of larval fat cells in adult Drosophila melanogaster. The Journal of experimental biology. 2007;210(Pt 6):956–63. Epub 2007/03/06. 10.1242/jeb.001586 . 17337708

[70] Tauchi-Sato K, Ozeki S, Houjou T, Taguchi R, Fujimoto T. The surface of lipid droplets is a phospholipid monolayer with a unique Fatty Acid composition. The Journal of Biological Chemistry. 2002;277(46):44507–12. Epub 2002/09/11. 10.1074/jbc.M207712200 . 12221100

[71] Katz SS, Shipley GG, Small DM. Physical chemistry of the lipids of human atherosclerotic lesions. Demonstration of a lesion intermediate between fatty streaks and advanced plaques. The Journal of clinical investigation. 1976;58(1):200–11. 10.1172/JCI108450 932206 PMC333171

[72] Small D. The Physical State of Lipids of Biological Importance: Cholesteryl Esters, Cholesterol, Triglyceride In: Blank M, editor. Surface Chemistry of Biological Systems. Advances in Experimental Medicine and Biology. 7: Springer US; 1970 p. 55–83.

[73] McIntosh AL, Storey SM, Atshaves BP. Intracellular Lipid Droplets Contain Dynamic Pools of Sphingomyelin: ADRP Binds Phospholipids with High Affinity. Lipids. 2010;45(6):465–77. 10.1007/s11745-010-3424-1. PMC3065392. 20473576 PMC3065392

[74] Mori M, Itabe H, Higashi Y, Fujimoto Y, Shiomi M, Yoshizumi M, et al Foam cell formation containing lipid droplets enriched with free cholesterol by hyperlipidemic serum. Journal of lipid research. 2001;42(11):1771–81. Epub 2001/11/21. . 11714846

[75] Robenek H, Robenek MJ, Troyer D. PAT family proteins pervade lipid droplet cores. Journal of lipid research. 2005;46(6):1331–8. 10.1194/jlr.M400323-JLR200. WOS:000229061100026. 15741656

[76] Pan M, Maitin V, Parathath S, Andreo U, Lin SX, St Germain C, et al Presecretory oxidation, aggregation, and autophagic destruction of apoprotein-B: a pathway for late-stage quality control. Proc Natl Acad Sci U S A. 2008;105(15):5862–7. Epub 2008/04/09. 10.1073/pnas.0707460104 ; PubMed Central PMCID: PMCPmc2311371. 18391222 PMC2311371

[77] Khaldoun SA, Emond-Boisjoly MA, Chateau D, Carriere V, Lacasa M, Rousset M, et al Autophagosomes contribute to intracellular lipid distribution in enterocytes. Mol Biol Cell. 2014;25(1):118–32. Epub 2013/11/01. 10.1091/mbc.E13-06-0324 ; PubMed Central PMCID: PMCPmc3873883. 24173715 PMC3873883

[78] Dupont N, Chauhan S, Arko-Mensah J, Castillo EF, Masedunskas A, Weigert R, et al Neutral lipid stores and lipase PNPLA5 contribute to autophagosome biogenesis. Current Biology. 2014;24(6):609–20. 10.1016/j.cub.2014.02.008 24613307 PMC4016984

[79] Singh R, Cuervo AM. Lipophagy: connecting autophagy and lipid metabolism. International Journal of Cell Biology. 2012;2012:282041 10.1155/2012/282041 22536247 PMC3320019

[80] Kaushik S, Cuervo AM. Degradation of lipid droplet-associated proteins by chaperone-mediated autophagy facilitates lipolysis. Nature cell biology. 2015;17(6):759–70. Epub 2015/05/12. 10.1038/ncb3166 ; PubMed Central PMCID: PMCPmc4449813. 25961502 PMC4449813

[81] Kaushik S, Rodriguez-Navarro JA, Arias E, Kiffin R, Sahu S, Schwartz GJ, et al Autophagy in hypothalamic AgRP neurons regulates food intake and energy balance. Cell metabolism. 2011;14(2):173–83. 10.1016/j.cmet.2011.06.008 21803288 PMC3148494

[82] Reggiori F, Tooze SA. Autophagy regulation through Atg9 traffic. The Journal of Cell Biology. 2012;198(2):151–3. 10.1083/jcb.201206119 22826119 PMC3410426

[83] Webber JL, Tooze SA. New insights into the function of Atg9. FEBS Letters. 2010;584(7):1319–26. 10.1016/j.febslet.2010.01.020 . 20083107

[84] Yang DS, Stavrides P, Saito M, Kumar A, Rodriguez-Navarro JA, Pawlik M, et al Defective macroautophagic turnover of brain lipids in the TgCRND8 Alzheimer mouse model: prevention by correcting lysosomal proteolytic deficits. Brain: a journal of neurology. 2014;137(Pt 12):3300–18. Epub 2014/10/02. 10.1093/brain/awu278 ; PubMed Central PMCID: PMCPmc4240291. 25270989 PMC4240291

[85] Russell RC, Yuan HX, Guan KL. Autophagy regulation by nutrient signaling. Cell research. 2014;24(1):42–57. 10.1038/cr.2013.166 24343578 PMC3879708

[86] Tracy K, Baehrecke EH. The role of autophagy in Drosophila metamorphosis. Current Topics in Developmental Biology. 2013;103:101–25. PubMed Central PMCID: PMC3896998. 23347517 10.1016/B978-0-12-385979-2.00004-6 PMC3896998

[87] Gronke S, Muller G, Hirsch J, Fellert S, Andreou A, Haase T, et al Dual lipolytic control of body fat storage and mobilization in Drosophila. PLOS Biology. 2007;5(6):e137 Epub 2007/05/10. 10.1371/journal.pbio.0050137 17488184 PMC1865564

[88] Wang S, Liu S, Liu H, Wang J, Zhou S, Jiang RJ, et al 20-hydroxyecdysone reduces insect food consumption resulting in fat body lipolysis during molting and pupation. Journal of Molecular Cell Biology. 2010;2(3):128–38. Epub 2010/05/01. 10.1093/jmcb/mjq006 . 20430856

[89] Grönke S, Mildner A, Fellert S, Tennagels N, Petry S, Müller G, et al Brummer lipase is an evolutionary conserved fat storage regulator in Drosophila. Cell metabolism. 2005;1(5):323–30. 10.1016/j.cmet.2005.04.003 16054079

[90] Hossain MS, Liu Y, Zhou S, Li K, Tian L, Li S. 20-Hydroxyecdysone-induced transcriptional activity of FoxO upregulates brummer and acid lipase-1 and promotes lipolysis in Bombyx fat body. Insect Biochem Mol Biol. 2013;43(9):829–38. Epub 2013/07/03. 10.1016/j.ibmb.2013.06.007 . 23811219

[91] Nezis IP. The selectivity and specificity of autophagy in Drosophila. Cells. 2012;1(3):248–62. Epub 2012/01/01. 10.3390/cells1030248 ; PubMed Central PMCID: PMCPmc3901107. 24710475 PMC3901107

[92] Govers R, Coster ACF, James DE. Insulin increases cell surface GLUT4 levels by dose dependently discharging GLUT4 into a cell surface recycling pathway. Mol Cell Biol. 2004;24(14):6456–66. WOS:000222490900030. 15226445 10.1128/MCB.24.14.6456-6466.2004 PMC434240

[93] Brand AH, Perrimon N. Targeted gene expression as a means of altering cell fates and generating dominant phenotypes. Development. 1993;118(2):401–15. Epub 1993/06/01. . 8223268 10.1242/dev.118.2.401

[94] Asha H, Nagy I, Kovacs G, Stetson D, Ando I, Dearolf CR. Analysis of Ras-induced overproliferation in Drosophila hemocytes. Genetics. 2003;163(1):203–15. Epub 2003/02/15. 12586708 10.1093/genetics/163.1.203 PMC1462399

[95] Kelso RJ, Buszczak M, Quinones AT, Castiblanco C, Mazzalupo S, Cooley L. Flytrap, a database documenting a GFP protein-trap insertion screen in Drosophila melanogaster. Nucleic Acids Research. 2004;32(Database issue):D418–20. Epub 2003/12/19. 10.1093/nar/gkh014 14681446 PMC308749

[96] Andres AJ, Thummel CS. Methods for quantitative analysis of transcription in larvae and prepupae. Methods in cell biology. 1994;44:565–73. Epub 1994/01/01. . 7535884 10.1016/s0091-679x(08)60932-2

[97] Rothstein EC, Nauman M, Chesnick S, Balaban RS. Multi-photon excitation microscopy in intact animals. Journal of Microscopy. 2006;222(Pt 1):58–64. Epub 2006/06/01. 10.1111/j.1365-2818.2006.01570.x 16734715 PMC1473170

