# Supplementary figures and images for: A Molecular Probe for the Detection of Polar Lipids in Live Cells
Source: PLoS One. 2016 Aug 23;11(8):e0161557. doi: 10.1371/journal.pone.0161557 (PMC4994960; doi:10.1371/journal.pone.0161557)

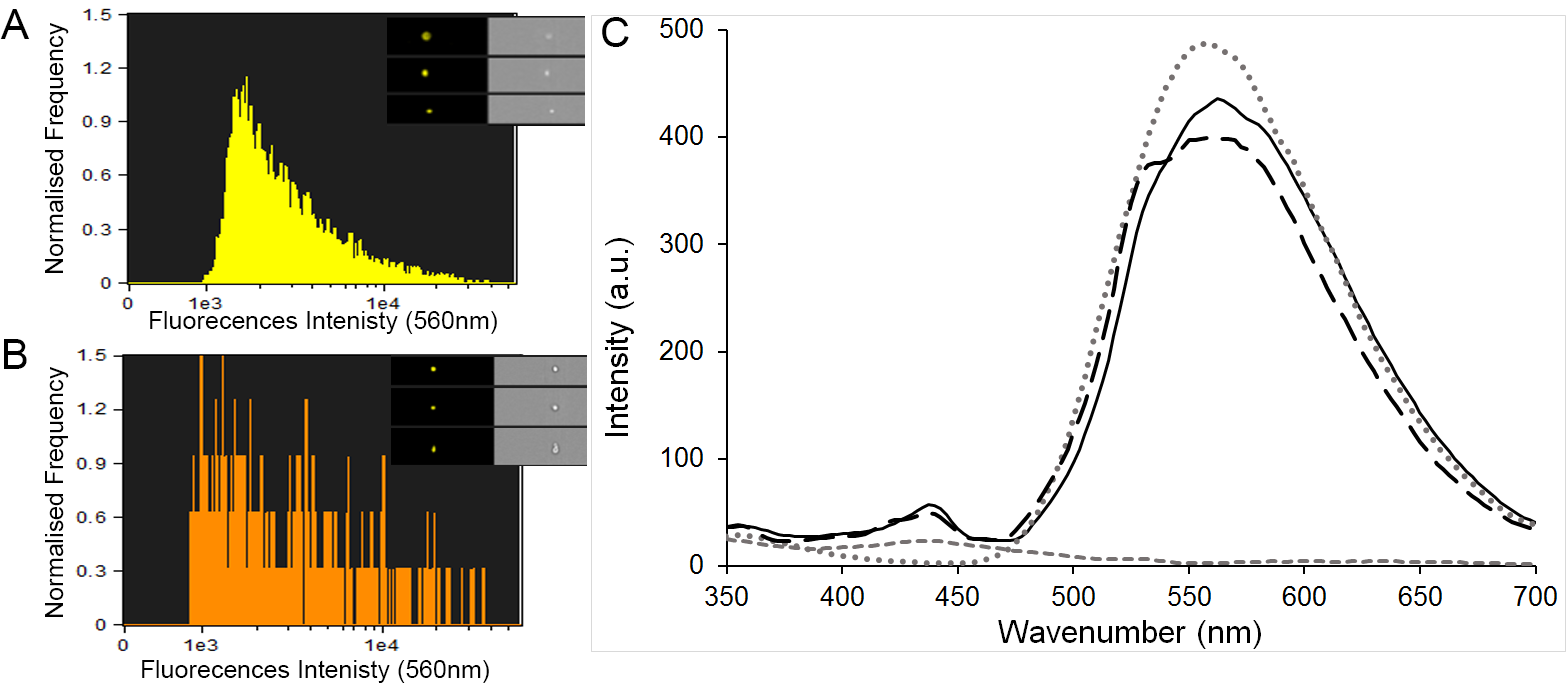

Supplement: S1 Fig — Histogram showing fluorescence intensity 560-595nm of DMPC vesicles (A) and DMPC and cholesterol vesicles (DMPC:cholesterol 8:1 molar ratio) (B) incubated with ReZolve-L1™ when excited at 405nm and representative fluorescence and transmitted lights images of vesicles by flow cytometry. (C) Fluoroscence spectra of liposomes alone (dashed grey line), ReZolve-L1™ alone (dotted grey line) and liposomes incubated with ReZolve-L1™ (black line; DMPC dashed line, DMPC and cholesterol solid line) when excited at 256nm. (TIF) [file pone.0161557.s001.tif]

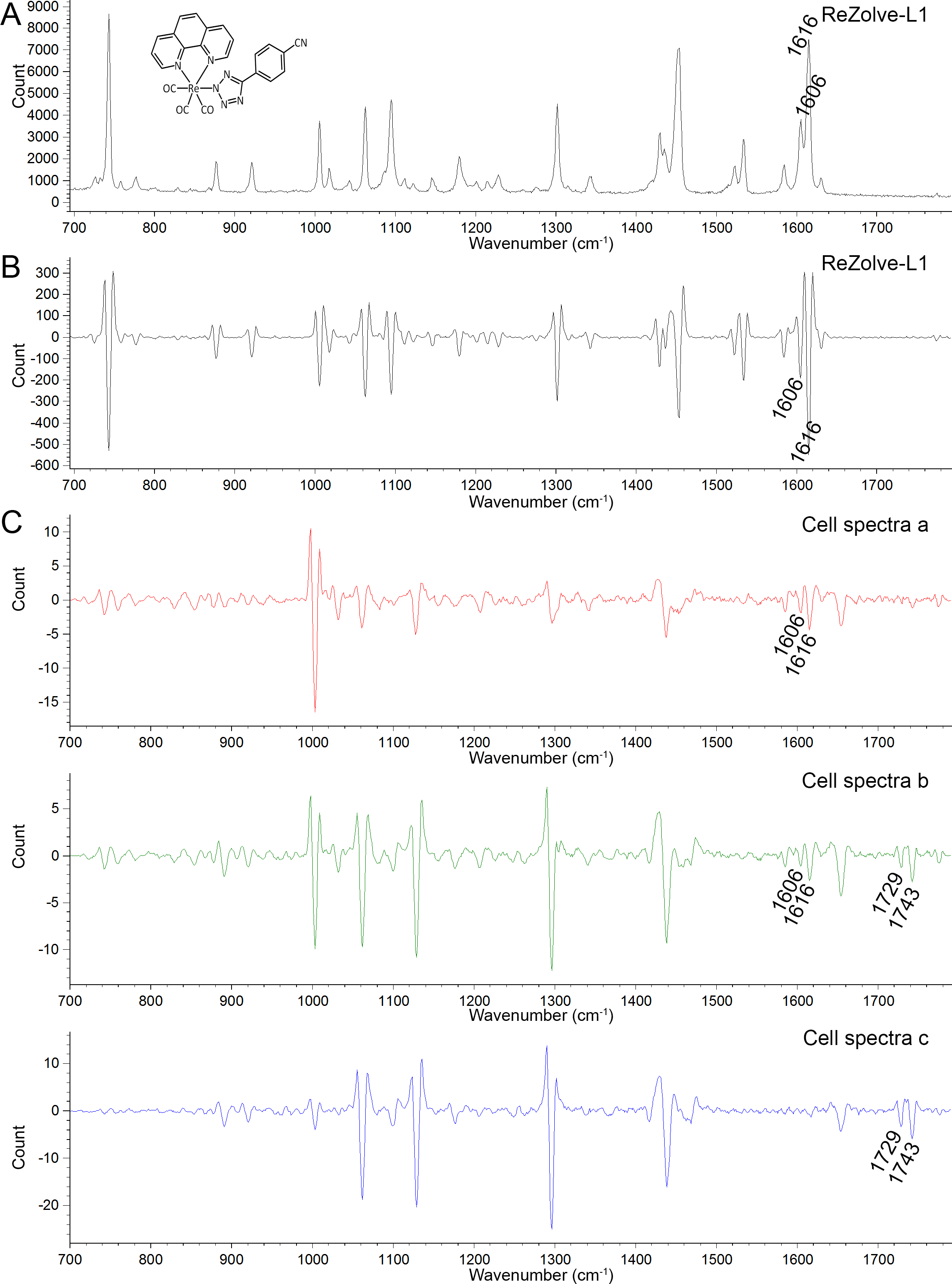

Supplement: S2 Fig — (A) Structure and Raman spectrum of ReZolve-L1™ and (B) the second derivative of this spectrum. (C) Second derivatives of Raman spectra taken from a ReZolve-L1™ stained 3T3 adipocytes from a region of high (a) medium (b) and low (c) ReZolve-L1™ presence. (TIF) [file pone.0161557.s002.tif]

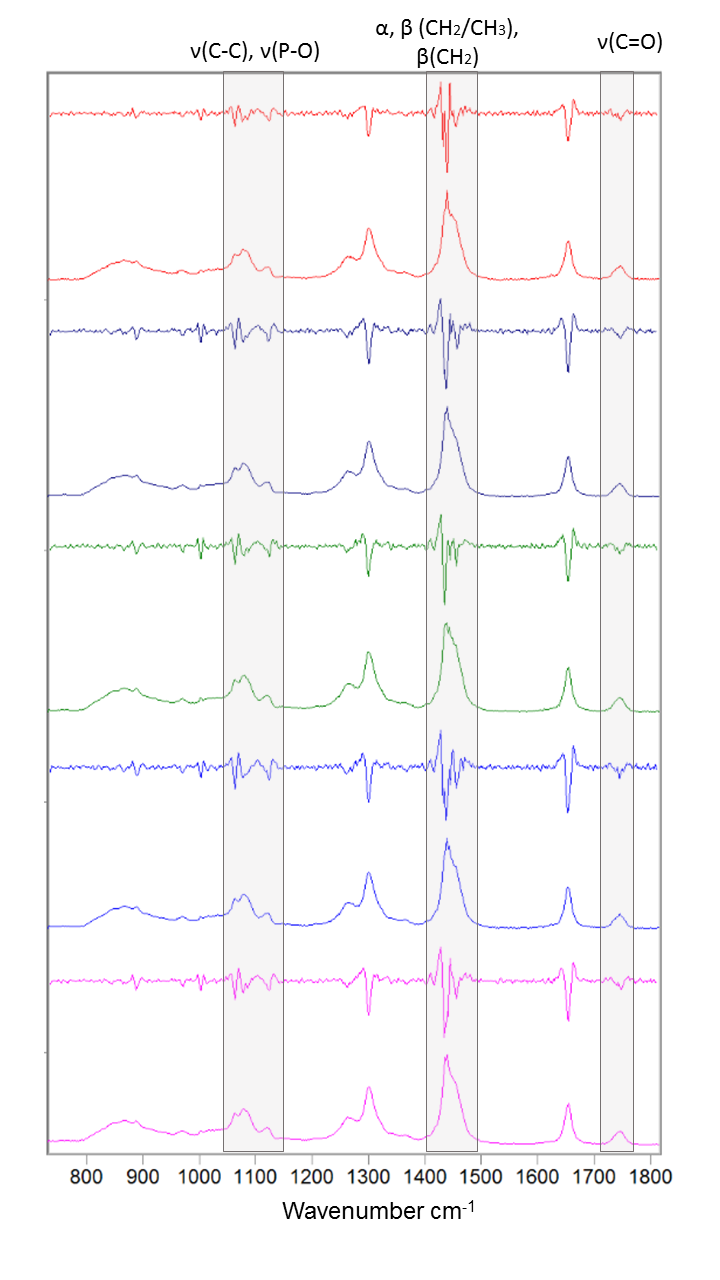

Supplement: S3 Fig — Representative Raman spectra from the lipid droplet core of -4 h PF larval fat body tissue. Corresponding second derivatives are presented above each of the five spectra. Important lipid regions are shaded and assigned above. (TIF) [file pone.0161557.s003.tif]

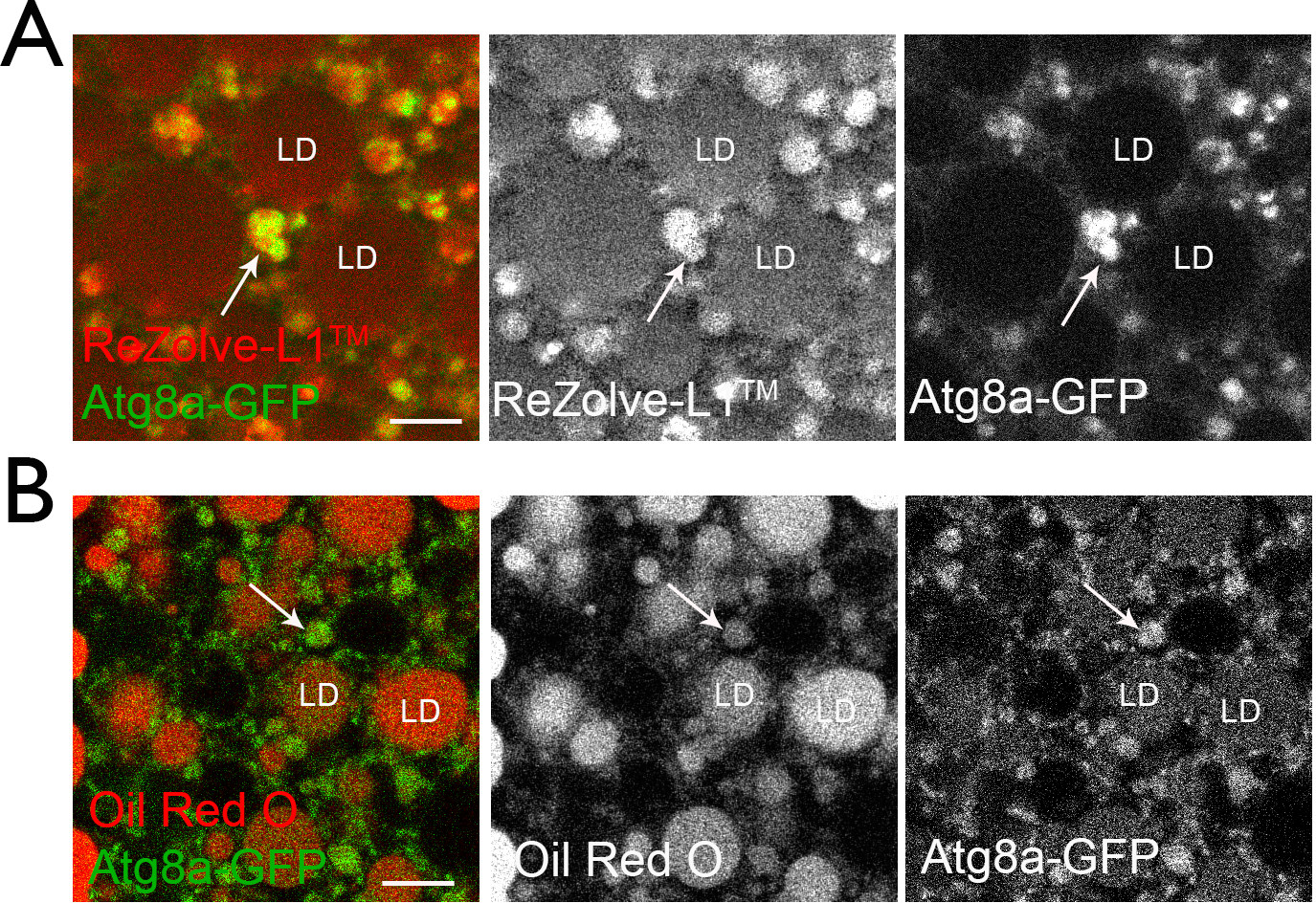

Supplement: S4 Fig — (A) Confocal micrographs of Drosophila fat body cells explanted from +2 h PF from pupae expressing Atg8a-GFP (green) and stained with ReZolve-L1™ (red). (B) Confocal micrographs of Drosophila fat body cells explanted from +2 h PF from pupae expressing Atg8a-GFP (green) and stained with Oil Red O (red). LD indicates lipid droplets arrows indicate co-location between ReZolve-L1 or Oil Red O and Atg8a-GFP autophagic compartments. Scale = 10 μm. (TIF) [file pone.0161557.s004.tif]
